# Genetic dissection of maize (Zea mays L.) chlorophyll content using multi-locus genome-wide association studies

**DOI:** 10.1186/s12864-023-09504-0

**Published:** 2023-07-10

**Authors:** Xuehang Xiong, Jianxin Li, Pingping Su, Haiyang Duan, Li Sun, Shuhao Xu, Yan Sun, Haidong Zhao, Xiaoyang Chen, Dong Ding, Xuehai Zhang, Jihua Tang

**Affiliations:** 1 National Key Laboratory of Wheat and Maize Crop Science, College of Agronomy, Henan Agricultural University, Zhengzhou, China; 2 The Shennong Laboratory, Zhengzhou, China

**Keywords:** Maize (Zea mays L.), Chlorophyll content, Single-locus GWAS, Multi-locus GWAS, High photosynthetic efficiency

## Abstract

**Background:**

The chlorophyll content (CC) is a key factor affecting maize photosynthetic efficiency and the final yield. However, its genetic basis remains unclear. The development of statistical methods has enabled researchers to design and apply various GWAS models, including MLM, MLMM, SUPER, FarmCPU, BLINK and 3VmrMLM. Comparative analysis of their results can lead to more effective mining of key genes.

**Results:**

The heritability of CC was 0.86. Six statistical models (MLM, BLINK, MLMM, FarmCPU, SUPER, and 3VmrMLM) and 1.25 million SNPs were used for the GWAS. A total of 140 quantitative trait nucleotides (QTNs) were detected, with 3VmrMLM and MLM detecting the most (118) and fewest (3) QTNs, respectively. The QTNs were associated with 481 genes and explained 0.29-10.28% of the phenotypic variation. Additionally, 10 co-located QTNs were detected by at least two different models or methods, three co-located QTNs were identified in at least two different environments, and six co-located QTNs were detected by different models or methods in different environments. Moreover, 69 candidate genes within or near these stable QTNs were screened based on the B73 (RefGen_v2) genome. *GRMZM2G110408* (*ZmCCS3*) was identified by multiple models and in multiple environments. The functional characterization of this gene indicated the encoded protein likely contributes to chlorophyll biosynthesis. In addition, the CC differed significantly between the haplotypes of the significant QTN in this gene, and CC was higher for haplotype 1.

**Conclusion:**

This study’s results broaden our understanding of the genetic basis of CC, mining key genes related to CC and may be relevant for the ideotype-based breeding of new maize varieties with high photosynthetic efficiency.

**Supplementary Information:**

The online version contains supplementary material available at 10.1186/s12864-023-09504-0.

## Background

Chlorophyll, which is an essential photosynthetic pigment in the chloroplasts of higher plants, is closely related to leaf photosynthesis and yield potential; it is critical for the accumulation of carbohydrates and contributes to complex processes mediating the acquisition of energy from light and electron transport [1–3]. In the *Arabidopsis thaliana* chlorophyll synthesis pathway, EIN3/EIL1 induces the expression of genes encoding protochlorophyllide oxidoreductase A and B, which cooperatively function with phytochrome-interacting factor 1 (PIF1) [4], thereby preventing seedling photo-oxidation and promoting cotyledon greening [5]. Furthermore, PIF1 can regulate the expression of *PORC* [6] and interact with gibberellin (GA)-regulated DELLA proteins [7] as well as a transposase-derived transcription factor (i.e., FHY3) to modulate chlorophyll biosynthesis [8]. Another study revealed that *BRAHMA* encodes the SWI2/SNF2 chromatin-remodeling ATPase that helps to regulate a novel mechanism underlying chlorophyll biosynthesis; compared with wild-type *A.thaliana* plants, RNA-interference transgenic seedlings of *BRAHMA* have a higher greening rate under light and accumulate less protochlorophyllide and reactive oxygen species [9]. The chlorophyll content (CC) is related to seedling development and survival [10], but it is also directly or indirectly related to leaf senescence and crop yield [11]. Earlier research on rice demonstrated that OSWRKY5 is a transcription factor that promotes leaf senescence via OsNAC2 [12], which affects abscisic acid-induced leaf senescence and the rice yield [11]. Similarly, the rice leaf CC and photosynthetic efficiency are closely associated with the accumulation of dry matter [13]. Moreover, there is a positive correlation between the grain CC and the grain filling rate [14]. Previous studies on the chlorophyll of Chinese cabbage [3], soybean [15], *Brassica napus* L. [16], and other plants [17] verified the importance of chlorophyll.

Maize is one of the most widely grown cereal crops worldwide. Accordingly, increasing maize productivity is crucial for agricultural development [18]. The ear leaf is one of the most important leaves of maize plants partly because of its close association with the yield [19]. Recent studies showed that increases in CC and the photosynthetic rate are critical for producing high maize yields [20, 21]. The Soil-Plant Analysis Development (SPAD) value, which may be used to represent the CC, can be determined using a rapid, accurate, and non-destructive measurement method involving the SPAD-502 chlorophyll meter [22]. Although several studies on maize chlorophyll-related genes were conducted recently [23, 24], the molecular mechanism of regulating chlorophyll remains to be elucidated. Therefore, an in-depth analysis of the genetic basis of the maize ear leaf CC is necessary for breeding new maize varieties with efficient photosynthetic activities and for increasing the maize yield.

A genome-wide association study (GWAS) can effectively reveal the genetic basis of complex quantitative traits according to linkage disequilibrium (LD). The advantages of linkage analyses over other methods include their higher throughput and greater resolution [25]. For example, *YIGE1*, which is an important gene for increasing maize ear growth and yield, was cloned following a GWAS [26]. Additionally, CC-related genes were mapped according to a GWAS [27, 28]. In another study, the rice flag leaf CC was determined and GWAS data were combined with high-density markers to detect several significant loci associated with chlorophyll-related traits in different rice subpopulations, including the locus for *Ghd7* [29]. The development of statistical methods has enabled researchers to design and apply various GWAS models, including single-locus model: MLM [25] and multi-locus models: MLMM [30], SUPER [31], FarmCPU [32], and BLINK [33]. Recently, 3VmrMLM was established as a novel MLM with three variance components for more efficient calculations [34]. This model is useful for identifying quantitative trait nucleotides (QTNs) and revealing QTN-by-environment interactions (QEIs) and QTN-by-QTN interactions.

In this study, the SPAD values of ear leaves from 290 maize inbred lines were determined in three environments and best linear unbiased prediction (BLUP) values were also calculated to represent the maize CC phenotype. A GWAS was performed using 1.25 million SNPs and six models (MLM, MLMM, SUPER, FarmCPU, BLINK, and 3VmrMLM) to elucidate the genetic basis of CC in maize. The results of this study will enrich our understanding of the molecular mechanism underlying the maize CC. The generated data may be used to further characterize the genes related to chlorophyll synthesis, while also providing the theoretical foundation for breeding maize plants with optimal light-use efficiency.

## Results

### Phenotypic variation

**Fig. 1:**
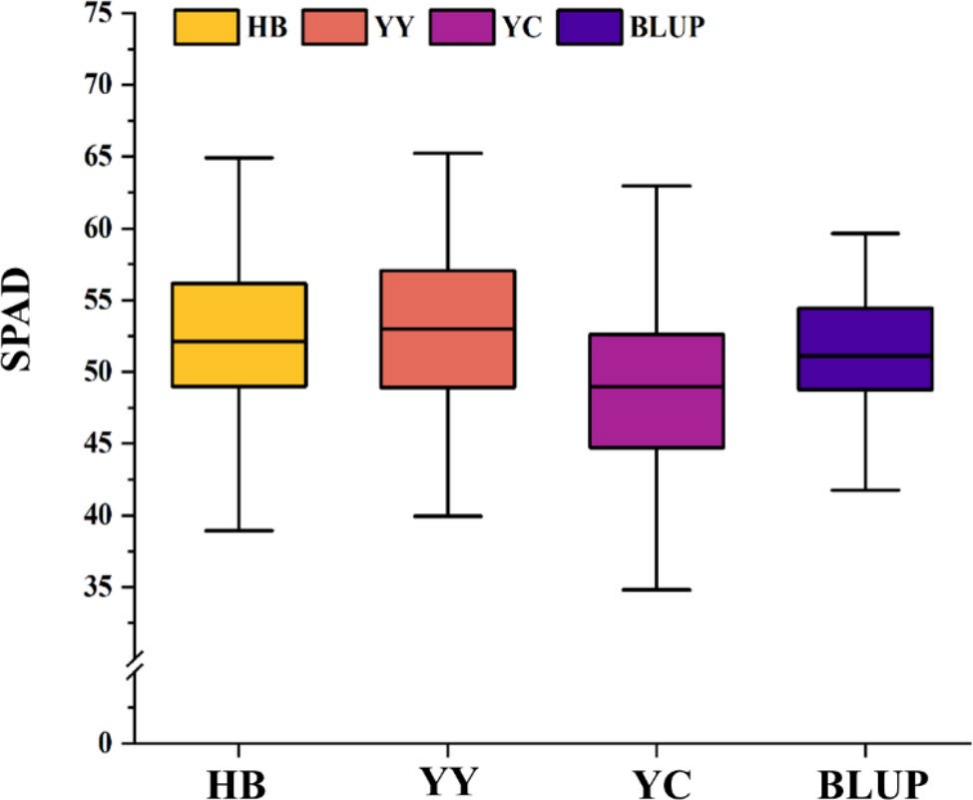
Variations in the SPAD values (CC) among the maize ear leaves in three environments (HB = Hebi, YY = Yuanyang, and YC = Yongcheng) and variations according to BLUP. The same abbreviations are used in the other figures. The black horizontal line indicates the median

**Fig. 2:**
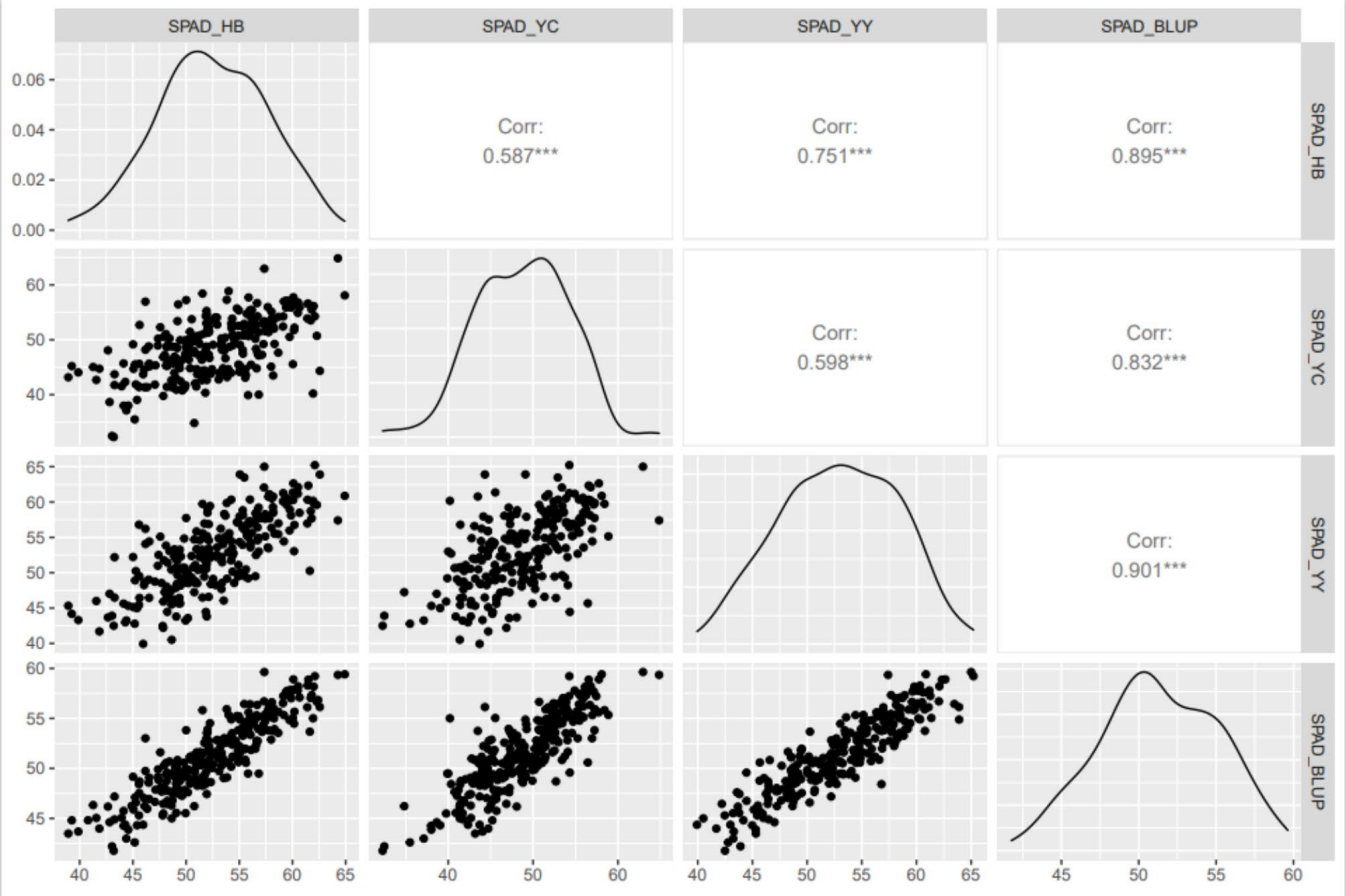
Pearson coefficients for the CC-related traits of maize lines in different environments. The lines were selected from an association mapping panel

The ear leaf Soil-Plant Analysis Development (SPAD) values [i.e., Chlorophyll Content (CC)] in Yuanyang (YY), Hebi (HB), and Yongchen (YC) at 10 days after pollination were analyzed. Additionally, the BLUP values were calculated and correlations were assessed. The index values in YC were lower than those in YY and HB, but similar values were obtained in YY and HB (Table 1; Fig. 1). Among the three environments (i.e., HB, YY, and YC) and Best Linear Unbiased Prediction (BLUP), the standard deviation in the SPAD value was highest for YC and YY (5.38), whereas the standard deviation was lowest for BLUP (3.83). The SPAD values were significantly correlated between any two environments (Fig. 2). Moreover, absolute value of skewness and kurtosis of the SPAD value for all environments and BLUP is less than 1 (Table 1) and it displayed a normal distribution (Figure S1), which implies CC follows a typical quantitative pattern that is controlled by numerous genes with small effects. The double-factor variance analysis revealed extremely significant genetic and environmental effects on CC. The genotype-by-environment interaction also had a significant effect (Table S1). According to the results, genetic factors influenced the maize CC more than environmental factors; the broad-sense heritability (0.86) was consistent with this observation (Table 1).

**Table 1:** Descriptive statistics of the CC (SPAD value) of maize in different environments

Trait	Environment	Range	Mean	sd.	Ske.	Kur.		*H* ^*2*^
SPAD	HB	38.93–64.91	52.48	5.14	-0.09	-0.40		0.86
	YC	32.28–64.85	48.74	5.38	-0.17	-0.07		
	YY	39.93–65.22	52.90	5.38	-0.09	-0.69		
	BLUP	41.76–59.64	51.33	3.83	-0.08	-0.58		

sd., standard deviation for the sample population

Ske., skewness (i.e., degree of asymmetry used to represent the relative mean)

Kur., kurtosis (i.e., peak value used to represent the dataset)

*H*^*2*^, broad-sense heritability

### Identification of the QTNs for the CC-related traits based on a GWAS

**Fig. 3:**
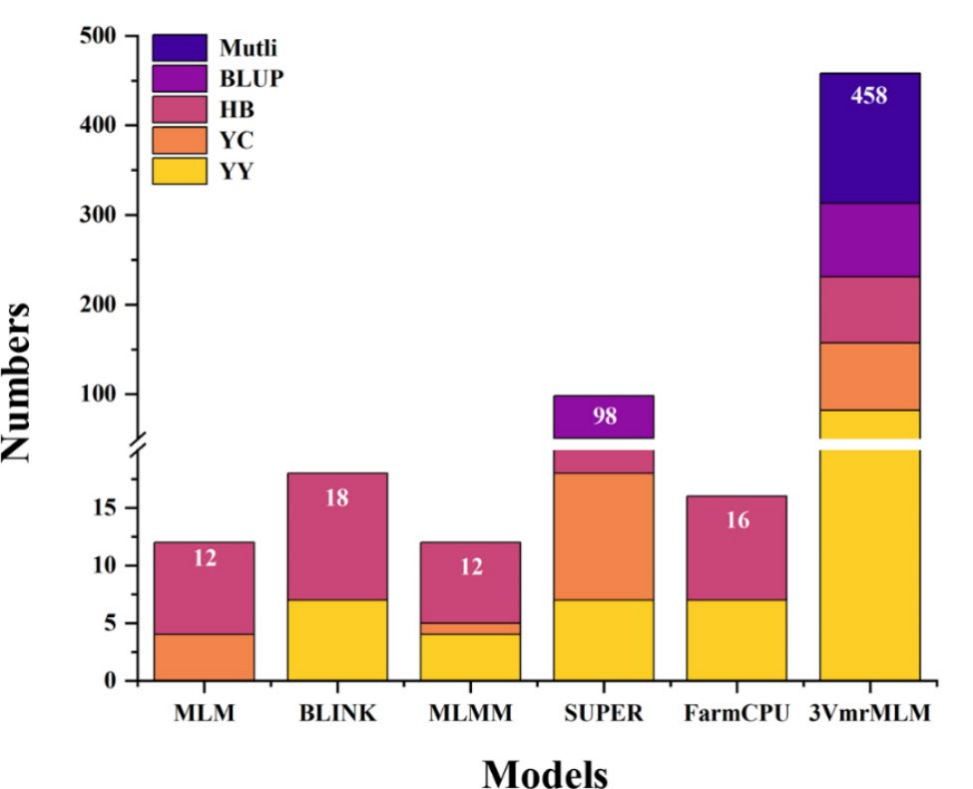
Number of candidate genes identified in different environments or by different models. “YY”, “YC”, “HB”, “BLUP” and “Mutli” indicated that the candidate genes identified in the environments of Yuanyang, Yongcheng, Hebi, Best linear unbiased prediction and by 3VmrMLM “Multi_env” method, respectively

A GWAS was performed using six models (MLM, BLINK, MLMM, SUPER, FarmCPU, and 3VmrMLM). The QQ plots of all of the models, with the exception of 3VmrMLM, reflected the relative reliability of the GWAS results (Figure S2). Using − log_10_(*p*-value) ≥ 5.75 or LOD score ≥ 3 as the significance threshold, we counted the QTNs for the six models in different environments. In addition to the “Single_env” method, the “Multi_env” method of 3VmrMLM was used to detect QTNs. The identified candidate genes were divided according to the models, environments, and methods. In YY, the BLINK, MLMM, SUPER, FarmCPU, and 3VmrMLM (“Single_env”) methods identified 7, 4, 7, 7, and 76 candidate genes, respectively. In YC, 4, 1, 11, and 81 candidate genes were detected using MLM, MLMM, SUPER, and 3VmrMLM. In HB, 8, 11, 7, 33, 9, and 74 candidate genes were revealed by MLM, BLINK, MLMM, SUPER, FarmCPU, and 3VmrMLM, respectively. For BLUP, 47 and 82 candidate genes were detected by SUPER and 3VmrMLM (“Single_env”), respectively, whereas 3VmrMLM (“Multi_env”) detected 145 candidate genes, including 21 genes associated with QEIs (Table S2, Fig. 3). The R^2^ values for the QTNs were 0.29–10.28, indicating they explained 0.29–10.28% of the phenotypic variation. The LOD scores for the QTNs detected using 3VmrMLM were 3.16–43.35 (Table S2). Thus, compared with the other models, SUPER and 3VmrMLM detected more candidate genes for the subsequent correlation analysis.

### Analysis of co-located QTNs

**Fig. 4:**
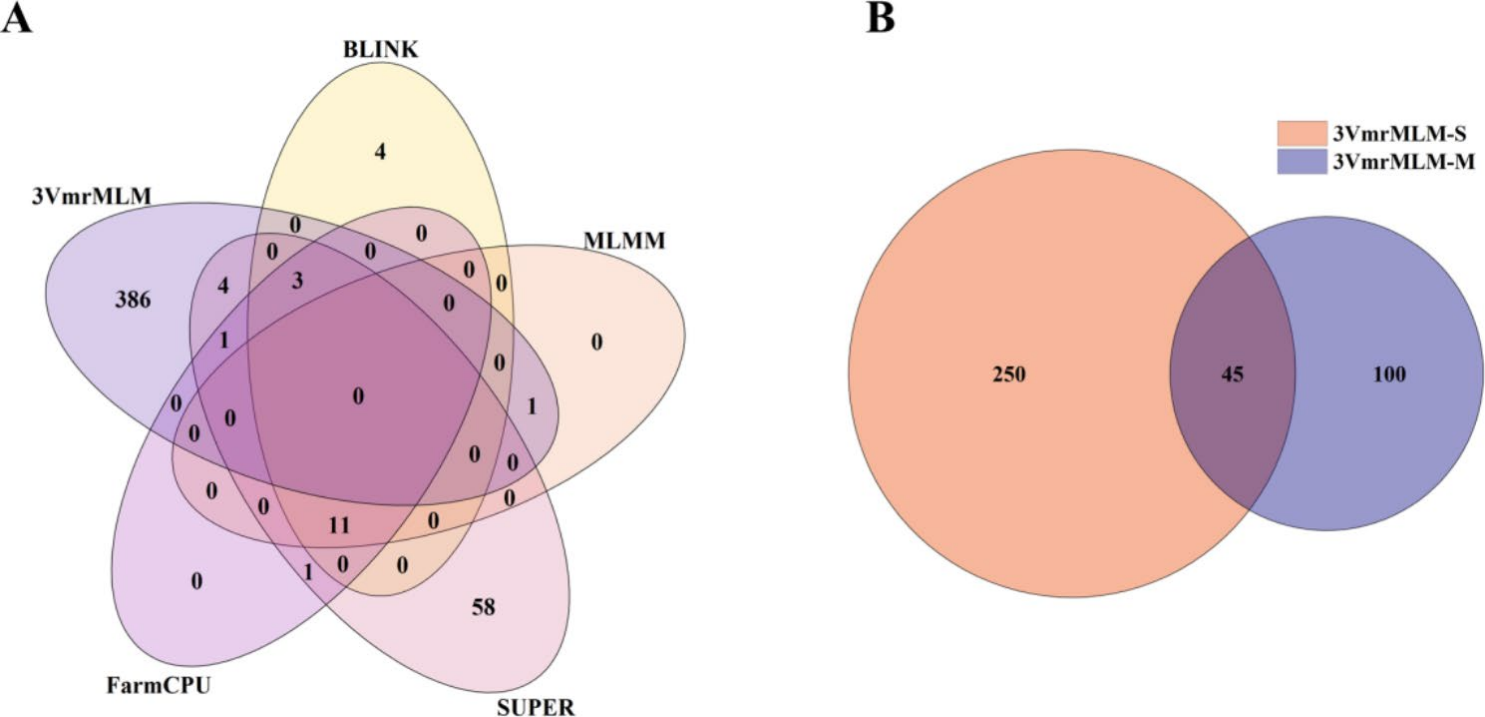
Candidate genes revealed by the models used for the GWAS. **(A)** Venn diagram of the number of co-located candidate genes detected by five models. **(B)** Venn diagram of the number of co-located candidate genes detected by the 3VmrMLM “Single_env” and “Multi_env” methods

We investigated 19 co-located QTNs across various models, methods, or environments. Out of these, four QTNs were detected within the same environment but using different models. Additionally, three QTNs were detected in different environments, but utilizing the same model. Furthermore, three QTNs were discovered in different environments and analyzed using different models. Three QTNs were found in different environments and analyzed using different methods. It is worth noting that six QTNs were identified in the same environment through the application of both the “Single_env” and “Multi_env” methods of 3VmrMLM. Moreover, three QTNs were detected in different environments using the two methods of 3VmrMLM (Table 2). For each QTN, we defined a 100 kb interval, encompassing 50 kb upstream and downstream, as the respective QTL region. It is important to highlight that two of the co-located QTNs were within the same QTL, while the remaining 17 QTNs were situated in distinct QTL regions. Out of these 18 QTL regions, two lacked identified candidate genes, However, the remaining 16 QTL regions contained a total of 69 candidate genes (Table 2). These candidate genes detected by each model were analyzed and summarized. In details, the candidate genes detected using MLM were not co-located with the candidate genes detected by the other five models. Furthermore, we perform a comprehensive evaluation of different models, with a specific emphasis on their efficacy in gene localization and 21 candidate genes are the co-located genes that identified at least two different models. Notably, SUPER, 3VmrMLM, and FarmCPU demonstrated superior performance and were therefore deemed to produce better results because they exhibited the highest number of co-located candidate genes (Fig. 4A). Additionally, the 3VmrMLM model (only this model) identified a total of 395 candidate genes. Specifically, 295 genes were identified using the “Single_env” method, while 145 genes were identified using the “Multi_env” method. Importantly, there were 45 candidate genes that were detected by both the “Single_env” and “Multi_env” methods of the 3VmrMLM model (Fig. 4B).

**Table 2:** Co-located QTNs and candidate genes as well as the related homologs

QTN	Chr.	Pos.	R^2^(%)	Model/Method	Env.	Sig.	LOD	*p*-value	Candidate genes	*Arabidopsis*/Oryza sativa homologs		Functional annotation	References
chr1.S_2694039	1	2,694,039	2.06	3VmrMLM-S	BLUP	SUG	6.085	1.20 × 10^− 7^	*GRMZM2G035377*	*AT5G22940 (F8H)*		a member of glycosyltransferase family 47	[70]
			1.47	3VmrMLM-M	ALL	SIG	16.89	1.16 × 10^− 18^	*GRMZM2G085691*	*AT5G03450*		Transducin/WD40 repeat-like superfamily protein	
									*GRMZM2G388684*	*AT2G36870 (XTH32)*		xyloglucan endotransglucosylase/hydrolase 32	[71]
									*GRMZM2G085827*	*AT3G11470*		4’-phosphopantetheinyl transferase superfamily	
									*GRMZM2G085872*	*AT4G31040 (DLDG1)*		Chloroplast membrane protein that modulates H + homeostasis	[72]
										*LOC_Os08g12780 (ARE1)*		chloroplast envelope membrane protein	[73]
									*GRMZM2G085885*	*AT5G35100 (CYP28)*		Cyclophilin-like peptidyl-prolyl cis-trans isomerase family protein	[74]
									*GRMZM2G086242*	*AT4G31040 (DLDG1)*		Chloroplast membrane protein that modulates H + homeostasis	[72]
										*LOC_Os08g12780 (ARE1)*		chloroplast envelope membrane protein	[73]
chr1.S_25147299	1	25,147,299	3.91	3VmrMLM-S	HB	SIG	8.856	1.70 × 10^− 10^	*GRMZM2G023364*	NA		NA	
			1.35	3VmrMLM-S	BLUP	SUG	3.577	4.94 × 10^− 5^	*GRMZM2G326869*	*AT2G29200 (PUM1)*		a member of the (APUM) proteins containing PUF domain	[75]
									*GRMZM2G376074*	*AT3G49600 (UBP26)*		Encodes a ubiquitin-specific protease	[76]
									*GRMZM2G077045*	*AT1G25580 (SOG1)*		Encodes suppressor of gamma response 1 (SOG1)	[77]
chr1.S_258002397	1	258,002,397	NA	BLINK	HB	NA	NA	2.97 × 10^− 10^	*GRMZM2G107120*	NA		NA	
			NA	MLMM	HB	NA	NA	1.48 × 10^− 6^	*GRMZM2G703998*	NA		NA	
			NA	SUPER	HB	NA	NA	1.70 × 10^− 7^	*GRMZM2G055037*	*AT5G45100 (BRG1)*		one of the BRGs (BOI-related gene)	[78]
			NA	FarmCPU	HB	NA	NA	6.92 × 10^− 7^	*GRMZM2G319281*	*AT3G50690*		Leucine-rich repeat (LRR) family protein	
									*GRMZM2G044762*	*AT3G46940 (DUT1)*		DUTP-PYROPHOSPHATASE-LIKE 1	[79]
									*GRMZM2G045314*	*AT4G34460 (AGB1)*		GTP binding protein beta 1	[80]
										*LOC_Os03g46650 (RGB1)*		guanine nucleotide-binding protein subunit beta-like	[40]
									*GRMZM2G046101*	*AT4G34480*		O-Glycosyl hydrolases family 17 protein	
chr1.S_282470596	1	282,470,596	NA	MLMM	YC	NA	NA	1.32 × 10^− 6^	*GRMZM5G837018*	*AT3G49650*		P-loop containing nucleoside triphosphate hydrolases superfamily protein	
			1.07	3VmrMLM-S	YC	SIG	7.483	4.36 × 10^− 9^					
chr2.S_218342905	2	218,342,905	NA	SUPER	BLUP	NA	NA	4.16 × 10^− 7^	*GRMZM2G453845*	*AT4G00690*		UB-like protease 1B	
			0.42	3VmrMLM-M	ALL	SIG	19.08	7.09 × 10^− 21^	*GRMZM2G151905*	*AT3G08960 (KA120)*		ARM repeat superfamily protein	[81]
									*GRMZM2G151975*	*AT1G09900*		Pentatricopeptide repeat (PPR-like) superfamily protein	
									*GRMZM2G151979*	NA		NA	
chr3.S_113480155	3	113,480,155	1.41	3VmrMLM-S	HB	SUG	4.371	7.24 × 10^− 6^	*GRMZM2G062080*	*AT5G05520*		Outer membrane OMP85 family protein	
			1.79	3VmrMLM-M	ALL	SIG	24.09	6.18 × 10^− 26^	*GRMZM2G395549*	*AT3G11130 (CHC1)*		CHC1 heavy chain subunit of clathrin	[82]
									*GRMZM2G093347*	*AT1G27450 (APT1)*		Adenosine phosphoribosyl transferase (E.C:2.4.2.7)	[41]
chr4.S_233763894	4	233,763,894	1.75	3VmrMLM-S	YC	SIG	7.795	2.08 × 10^− 9^	*GRMZM2G056867*	*AT1G51130 (NSE4A)*		δ-kleisin component of the SMC5/6 complex	[83]
			1.73	3VmrMLM-S	HB	SIG	7.356	5.88 × 10^− 9^	*GRMZM2G056920*	*AT3G12130 (KHZ1)*		a CCCH zinc-finger and KH domain protein (KHZ1)	[39]
			3.49	3VmrMLM-S	BLUP	SIG	15.69	1.91 × 10^− 17^	*AC204231.3_FG010*	NA		NA	
			0.65	3VmrMLM-M	ALL	SIG	12.33	4.88 × 10^− 14^	*GRMZM2G063909*	*AT2G44350 (ATCS)*		mitochrondrion targeted citrate synthase	[84]
chr5.S_70070643	5	70,070,643	2.96	3VmrMLM-S	BLUP	SIG	7.437	4.85 × 10^− 9^	*GRMZM2G044348*	*AT2G03120 (AtSPP)*		homologous to Signal Peptide Peptidases (SPP)	[85]
			0.80	3VmrMLM-M	ALL	SIG	8.279	6.64 × 10^− 10^		*LOC_Os02g02530 (OsSPP1)*		signal peptide peptidase 1-like	[86]
									*GRMZM2G098301*	*AT1G42540 (GLR3.3)*		member of Putative ligand-gated ion channel subunit family	[87]
									*GRMZM2G098370*	*AT5G17310 (UGP2)*		UDP-glucose pyrophosphorylase	[88]
										*LOC_Os02g02560 (OsUgp2)*		UTP–glucose-1-phosphate uridylyltransferase	[89]
									*GRMZM2G098420*	*AT5G17290 (ATG5)*		Autophagy protein ATG5	[90]
									*GRMZM2G098438*	*AT5G64470*		trichome birefringence-like protein	
									*GRMZM2G156924*	NA		NA	
									*GRMZM2G156824*	*AT4G35600 (CST)*		a receptor-like cytoplasmic kinase that acts	[91]
									*GRMZM2G456917*	*AT3G02570 (PMI1)*		protein with phosphomannose isomerase activity.	[92]
chr5.S_73045639	5	73,045,639	NA	BLINK	YY	NA	NA	7.86 × 10^− 7^	*GRMZM2G029065*	*AT4G12080 (AHL1)*		AT-hook motif nuclear-localized protein 1	[93]
			NA	MLMM	YY	NA	NA	7.69 × 10^− 7^	*GRMZM2G029087*	*AT5G47120 (ATBI-1)*		a homolog of mammalian Bax inhibitor 1 (BI-1)	[94]
			NA	FarmCPU	YY	NA	NA	7.86 × 10^− 7^	*GRMZM2G029879*	*AT2G44740*		cyclin p4	
			NA	SUPER	YY	NA	NA	1.64 × 10^− 7^	*GRMZM2G030013*	*AT4G26000 (PEPPER)*		a polypeptide with K-homology (KH) RNA-binding modules	[95]
			NA	SUPER	HB	NA	NA	1.16 × 10^− 6^					
			NA	SUPER	BLUP	NA	NA	1.34 × 10^− 6^					
chr5.S_209582673	5	209,582,673	NA	BLINK	YY	NA	NA	1.14 × 10^− 6^	*GRMZM2G046885*	*AT2G22540 (SVP)*		a nuclear protein that acts as a floral repressor	[96]
										*LOC_Os02g52340*		MADS-box transcription factor 22-like (OsMADS22)	[97]
			NA	SUPER	YY	NA	NA	2.74 × 10^− 7^	*GRMZM2G012178*	*AT4G33945*		ARM repeat superfamily protein	
			NA	SUPER	HB	NA	NA	3.47 × 10^− 7^	*GRMZM2G465430*	*AT5G47120 (ATBI-1)*		a homolog of mammalian Bax inhibitor 1 (BI-1),	[94]
			NA	SUPER	BLUP	NA	NA	1.14 × 10^− 6^					
			NA	FarmCPU	YY	NA	NA	1.14 × 10^− 6^					
			4.63	3VmrMLM-S	YY	SIG	43.35	2.52 × 10^− 45^					
chr5.S_215364939	5	215,364,939	NA	SUPER	HB	NA	NA	7.55 × 10^− 7^	*GRMZM5G897740*	*AT5G42223*		a defensin-like (DEFL) family protein	
			NA	SUPER	BLUP	NA	NA	6.43 × 10^− 7^	*GRMZM2G079013*	*AT2G32700 (LUH)*		a WD40 repeat and LUFS domain containing protein	[98]
									*GRMZM2G078887*	*AT3G07090*		Interacts with C3H59 via its WD40 domain and C-terminal region	
									*GRMZM2G376595*	*AT1G04010 (PSAT1)*		aphosphoserine aminotransferase	[99]
chr7.S_170643809	7	170,643,809	1.37	3VmrMLM-S	BLUP	SIG	8.732	2.28 × 10^− 10^	*GRMZM2G436082*	*AT2G41770 (STL1)*		cellulose synthase complexes	[100]
			0.54	3VmrMLM-M	ALL	SIG	13.75	1.75 × 10^− 15^	*GRMZM2G133400*	*AT5G13720*		Uncharacterized protein family	[101]
									*GRMZM2G133392*	*AT5G27320 (GID1C)*		a gibberellin (GA) receptor	[102]
									*GRMZM2G133358*	*AT3G60910*		S-adenosyl-L-methionine-dependent methyltransferases superfamily protein	
									*GRMZM2G433767*	*AT3G63470*		serine carboxypeptidase-like 40	
									*GRMZM2G133275*	*AT2G28420*		Vicinal oxygen chelate (VOC) superfamily member	
										*LOC_Os07g46360 (OsGly I)*		glyoxalase family protein	[103]
chr9.S_110657959	9	110,657,959	NA	SUPER	HB	NA	NA	2.53 × 10^− 7^	*GRMZM2G110408*	*AT2G30950 (VAR2)*		Metalloprotease	[37]
			NA	SUPER	BLUP	NA	NA	1.74 × 10^− 6^	*GRMZM2G110646*	*AT1G68000 (PIS1)*		phosphatidylinositol synthase 1	[104]
			NA	FarmCPU	HB	NA	NA	1.27 × 10^− 6^					
chr9.S_110659989	9	110,659,989	NA	SUPER	HB	NA	NA	9.31 × 10^− 7^	*GRMZM2G110408*	*AT2G30950 (VAR2)*		Metalloprotease	[37]
			NA	SUPER	BLUP	NA	NA	1.74 × 10^− 6^	*GRMZM2G110646*	*AT1G68000 (PIS1)*		phosphatidylinositol synthase 1	[104]
chr9.S_111331253	9	111,331,253	3.76	3VmrMLM-S	HB	SUG	5.44	5.59 × 10^− 7^					
			2.18	3VmrMLM-S	BLUP	SUG	3.612	4.54 × 10^− 5^					
			2.31	3VmrMLM-M	ALL	SIG	14.75	1.72 × 10^− 16^					
chr9.S_152202624	9	152,202,624	1.94	3VmrMLM-S	BLUP	SUG	5.023	1.51 × 10^− 6^	*GRMZM2G178072*	*AT3G24010*		a member of the Inhibitor of Growth family homeodomain proteins	
			0.88	3VmrMLM-M	ALL	SIG	8.909	1.50 × 10^− 10^	*GRMZM5G865819*	*AT2G20370 (UMR3)*		a xyloglucan galactosyltransferase	[105]
									*GRMZM2G137779*	*AT2G20370 (UMR3)*		a xyloglucan galactosyltransferase	[105]
chr10.S_3188051	10	3,188,051	2.91	3VmrMLM-S	BLUP	SIG	10.37	4.85 × 10^− 12^	*GRMZM2G356817*	*AT3G14470 (LRR4)*		NB-ARC domain-containing disease resistance protein	[106]
			0.83	3VmrMLM-M	ALL	SIG	12.36	4.55 × 10^− 14^	*GRMZM2G356839*	NA		NA	
									*GRMZM2G058481*	*AT3G08600*		transmembrane protein, putative	
									*GRMZM2G058531*	*AT3G08570*		Phototropic-responsive NPH3 family protein	
										*LOC_Os02g35970 (CPT1)*		coleoptile phototropism protein 1-like	[107]
									*GRMZM5G862857*	NA		NA	
									*GRMZM2G058595*	*AT2G30520 (RPT2)*		a phototropin-interacting NRL protein	[108]
chr10.S_12754330	10	12,754,330	3.59	3VmrMLM-S	YY	SIG	15.03	8.91 × 10^− 17^	*GRMZM2G137407*	*AT5G58380 (SIP1)*		a CBL-interacting protein kinase	[109]
			4.07	3VmrMLM-S	BLUP	SIG	13.53	2.94 × 10^− 15^		*LOC_Os12g02200 (OsCIPK14)*		CBL-interacting protein kinase 14-like	[110]
			2.23	3VmrMLM-M	ALL	SIG	30.22	4.06 × 10^− 32^	*GRMZM2G063394*	*AT1G76390 (PUB43)*		Plant U-box type E3 ubiquitin ligase	[111]
									*GRMZM5G802395*	NA		NA	
chr10.S_28506964	10	28,506,964	NA	MLMM	YY	NA	NA	1.06 × 10^− 6^					
			10.28	MLM	YY	NA	NA	1.38 × 10^− 6^					

Chr., chromosome; Pos., position; Env., environment; Sig., significance; 3VmrMLM-S, 3VmrMLM “Single_env” method; 3VmrMLM-M, 3VmrMLM “Multi_env” method; SIG, significant QTN; SUG, suggested QTN; and NA, no result, no homologous gene, or unknown function

### Candidate gene analysis

**Fig. 5:**
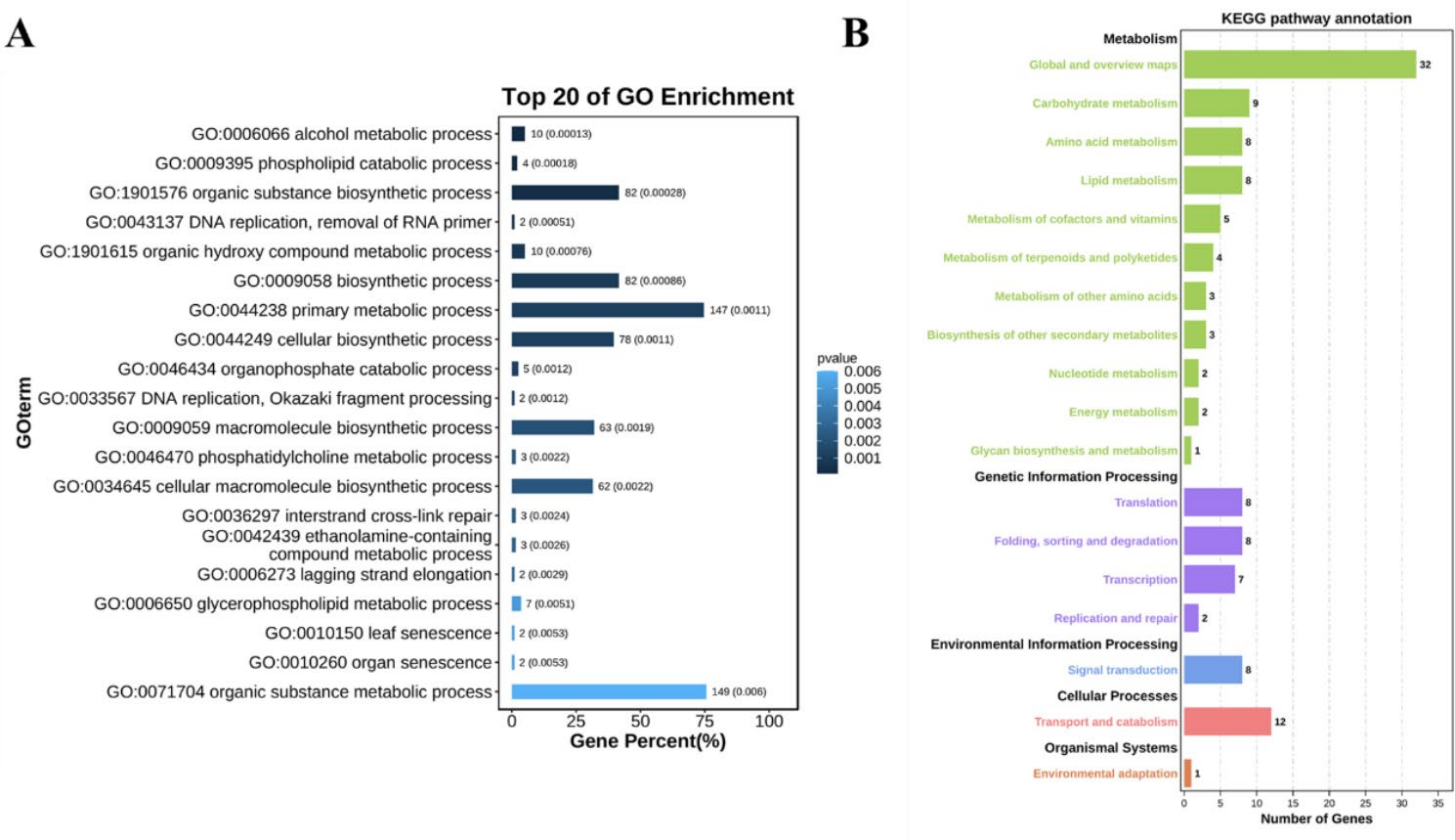
Enriched GO terms and KEGG pathways among the candidate genes. **(A)** Enriched GO terms in the biological process. **(B)** Enriched KEGG pathways

The candidate genes identified in Table S2 were categorized into two groups: those with functional annotations and those without functional annotations. We performed Gene Ontology (GO) and Kyoto Encyclopedia of Genes and Genomes (KEGG) enrichment analyses specifically for the genes with functional annotations. The top 20 GO terms related to biological processes and KEGG pathways were determined, revealing that the candidate genes were predominantly enriched in metabolic processes (Fig. 5). It has been established in previous studies that chlorophyll serves as the primary pigment for photosynthesis. Photosynthesis, being the basis and source of plant metabolism, suggests a close relationship between plant metabolism and chlorophyll [35, 36]. Taking into account the candidate genes located within the co-located QTNs, we conducted a detailed examination and identified corresponding homologs in *A. thaliana* or rice to further characterize the functional aspects of these candidate genes (Table 2). Based on the aforementioned findings, it is crucial to analyze the key candidate genes within the co-located QTLs. These genes hold significant potential for further research and exploration.

One of the candidate genes, *GRMZM2G110408*, was associated with two co-located QTNs (chr9.S_110657959 and chr9.S_110659989). This gene encodes a nucleoside triphosphatase involved in chlorophyll biosynthesis. In *Arabidopsis thaliana*, its homolog has been reported to encode a metalloproteinase that contributes to thylakoid membrane biogenesis [37] and aids in the repair of Photosystem II (PSII) after photoinhibition-related damage. The expression levels of this gene (both transcript and protein levels) increase in response to light, we named it as *ZmCCS3*. Among the annotated candidate genes, two genes (*GRMZM2G376595* and *GRMZM2G098420*) were found to be associated with leaf senescence (GO:0010150, *p*-value = 0.0052) (Fig. 5A), a process involving changes to chlorophyll [38]. *GRMZM2G098420*, co-located by both the “Single_env” and “Multi_env” methods of 3VmrMLM. In *Arabidopsis thaliana*, its homolog encodes an autophagy protein 5, which is part of the Atg12–Atg5–Atg16 complex (cellular component) (Table S3). This suggests its involvement in chlorophyll degradation. In contrast, *GRMZM2G376595* encodes a phosphatidylcholine-sterol O-acyltransferase. Another candidate gene, *GRMZM2G056920*, was co-located in three environments (YC, HB, and BLUP) according to the “Single_env” method and the “Multi_env” method. The LOD scores of the associated QTNs were relatively high (ranging from 7.36 to 15.69). This gene encodes a protein involved in the construction of the cell wall structure. A previous study indicated that the overexpression of its *Arabidopsis thaliana* homolog, *AT3G12130* (*KHZ1*), significantly promotes leaf senescence [39]. The gene *GRMZM2G045314*, co-located by four models (BLINK, MLMM, SUPER, FarmCPU), encodes a GTP-binding protein 2. In rice, its homolog is involved in controlling grain development and the grain filling process, directly affecting yield. This suggests that further research on this gene could be valuable for optimizing yield [40]. *GRMZM2G093347*, co-located by both the “Single_env” and “Multi_env” methods of 3VmrMLM, encodes adenine Adenine phosphoribosyl transferase 1 (APT1). An *Arabidopsis thaliana* mutant lacking *ATP1* activity exhibited higher chlorophyll content (compared to the wild-type leaf chlorophyll content), suggesting that this gene may be associated with chlorophyll accumulation [41].

Apart from the comprehensive analysis of the co-located candidate genes, we observed that 3VmrMLM outperformed the other models in terms of detecting QTNs and identifying more candidate genes associated with CC. For instance, GO analysis revealed genes enriched in protein localization within chloroplasts (GO:0072598, p-value = 0.0437) (Table S3), one such gene, *GRMZM5G839422*, was functionally annotated as encoding an inner membrane protein. In an earlier study, a mutant carrying a mutated *ALB3* gene (homologous to *GRMZM5G839422*) exhibited abnormal chloroplasts and a lower CC compared to the wild-type control [42]. The *A. thaliana* homolog of *GRMZM2G005848* (*ARC5*) encodes a chloroplast division protein. Mutations in this gene lead to a decrease in the number and size of chloroplasts in mesophyll cells [43]. In cucumbers, the homolog of this gene causes the cucumber peel to appear light green [44]. The functional annotations of this gene include biological processes related to chloroplast fission (GO: 0010020, *p*-value = 0.0240) and organization (GO:0009658, *p*-value = 0.0431) (Table S3). By searching for homologous genes in *Arabidopsis* and rice, some interesting discoveries were made. *GRMZM2G017077* appears to affect chloroplasts and chlorophyll synthesis. The *A. thaliana* homolog, *VIPP1*, is a multifunctional protein in chloroplasts with important effects on the envelope [45]. It is also involved in the maintenance of photosynthetic membranes [46]. The gene *GRMZM2G135283* falls within the QTL interval of the QTN (chr1.S_274280041, P-value = 1.18 × 10^− 12^) that located on chromosome 1 was detected in YC by 3VmrMLM model. Its rice homolog, *OsSHM1*, encodes a serine hydroxymethyltransferase. The CC of the *OsSHM1* mutant is significantly lower than that of the wild-type control and the mutant seedlings exhibit a less green phenotype compared to the wild-type seedlings during early growth and development [47]. The rice homolog of *GRMZM2G171444* encodes a chloroplast precursor, while the *A. thaliana* homolog encodes a chloroplast envelope and matrix protein that influences chlorophyll biosynthesis. However, further investigation and characterization of *GRMZM2G171444* are required. Based on the enriched GO terms, KEGG pathways, co-localization results and earlier studies on the homologs of the candidate genes, we selected 11 candidate genes. Among them, the QTN associated with *GRMZM2G005848* and *GRMZM2G098420* were located approximately 20 kb downstream of the two genes, while the QTN of the other nine genes were located within the gene regions. These genes show significant potential for further research into their association with CC.

### Analysis of candidate gene expression patterns

**Fig. 6:**
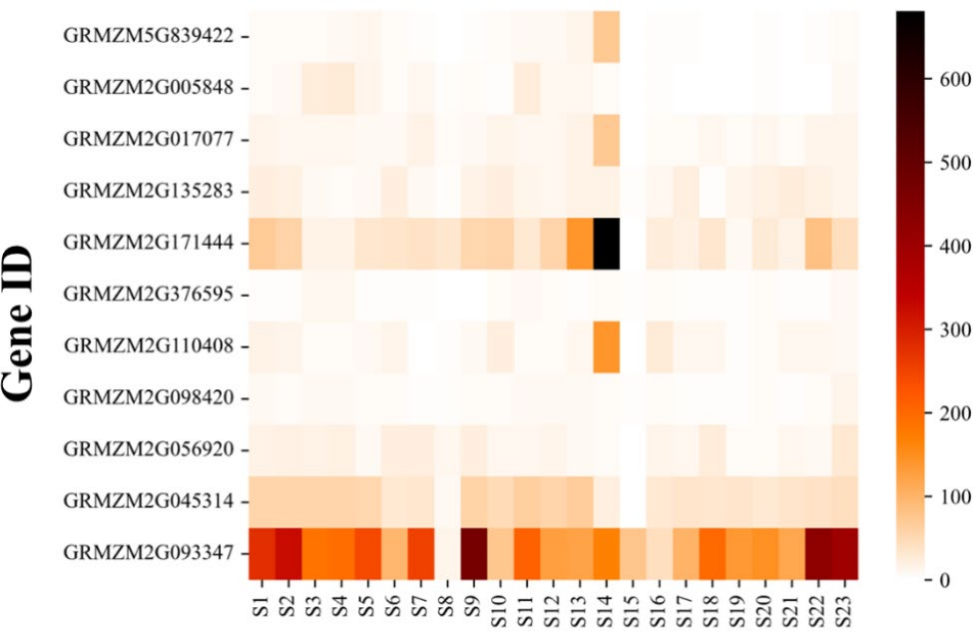
Heatmap of the expression profiles of key candidate genes. S1: 6–7_Internode, S2: 7–8_Internode, S3: Ear_Primordium_2–4_mm, S4: Ear_Primordium_6–8_mm, S5: Embryo_20_DAP, S6: Embryo_38_DAP, S7: Endosperm_12_DAP, S8: Endosperm_Crown_27_DAP, S9: Female_Spikelet_Collected_on_Day_as_Silk, S10: Germinated_Kernels_2_DAI, S11: Leaf_Zone_1_Symmetrical, S12: Leaf_Zone_2_Stomatal, S13: Leaf_Zone_3_Growth, S14: Mature_Leaf_8, S15: Mature_Pollen, S16: Pericarp_Aleurone_27_DAP, S17: Primary_Root_5_Days, S18: Root_Cortex_5_Days, S19: Elongation_Zone_5_Days, S20: Root_Meristem_Zone_5_Days, S21: Secondary_Root_7–8_Days, S22: Silk, S23: Vegetative_Meristem_16–19_Days

We examined the expression profiles of the 11 selected candidate genes in different B73 tissues and constructed a heatmap of the FPKM-based expression levels (Fig. 6). Earlier research confirmed CC is closely related to leaf photosynthesis [48] and directly or indirectly affects leaf senescence [11]. Therefore, we focused on the candidate genes highly expressed in the leaf tissues (S11–S14), especially the mature leaves (S14). The *GRMZM2G171444* expression level was significantly higher in S14 than in the other tissues. Moreover, *GRMZM2G171444* was the most highly expressed candidate gene in mature leaves (Fig. 6, Table S5). However, *GRMZM5G839422*, *GRMZM2G110408*, and *GRMZM2G017077* were also highly expressed in S14. The *GRMZM2G093347* expression level was high in all tissues. In contrast, the other genes were expressed at relatively low levels in the leaves. Overall, the analysis of candidate gene expression indicated that *GRMZM2G171444* and *ZmCCS3* may be related to CC and involved in chlorophyll synthesis, but *GRMZM5G839422* and *GRMZM2G017077* may also influence CC.

### Haplotype analysis of candidate genes

**Fig. 7:**
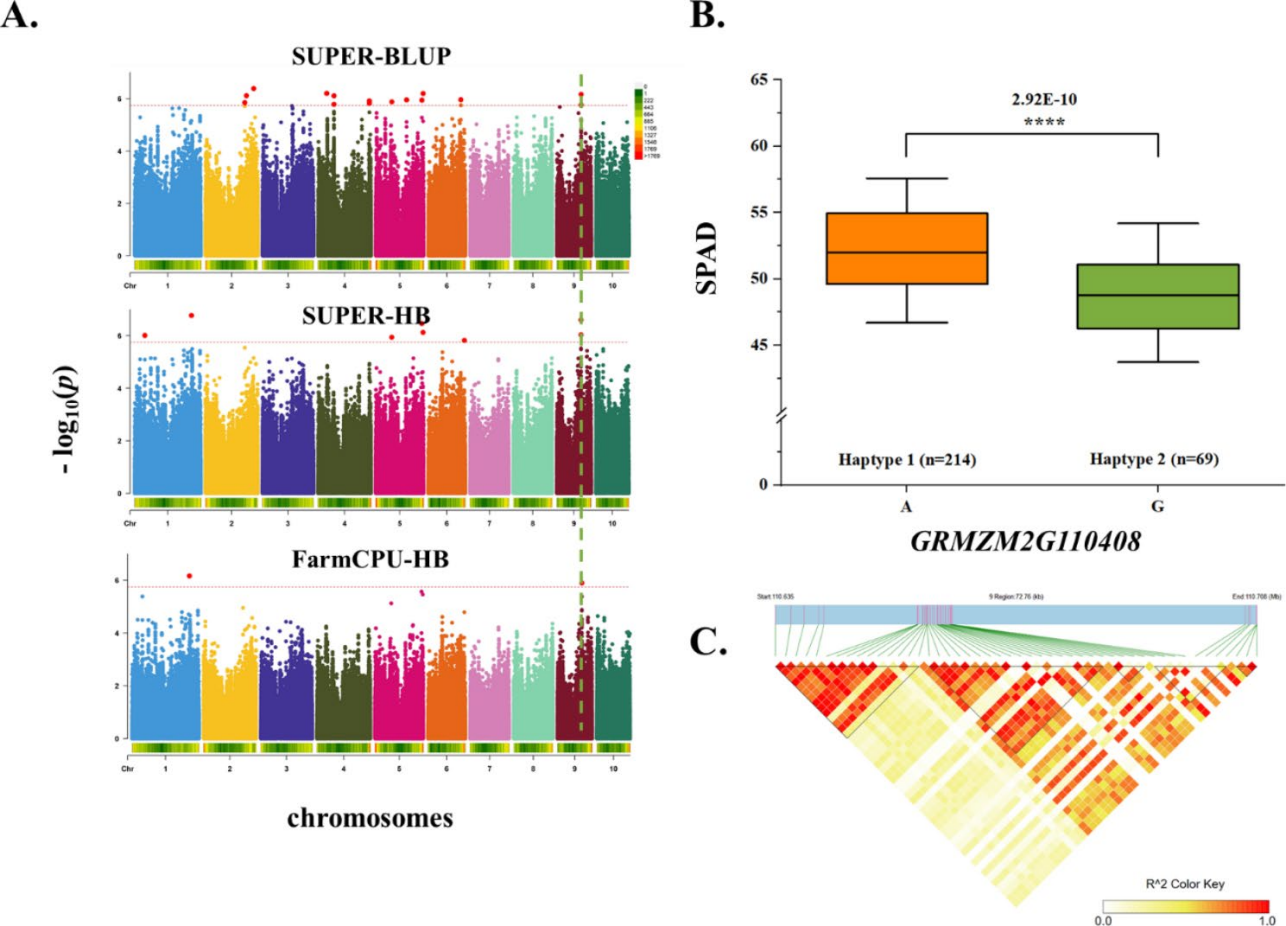
*GRMZM2G110408 (ZmCCS3)* affects CC-related traits. **(A)** Manhattan plot of the SPAD values for BLUP and HB. The line represents the threshold − log_10_(*p*-value) ≥ 5.75 (*p* ≤ 1.79 × 10^− 6^). **(B)** Differences in the CC-related traits of haplotypes 1 and 2. **(C)** R^2^ values for all SNPs in the QTL of the significant QTN

**Fig. 8:**
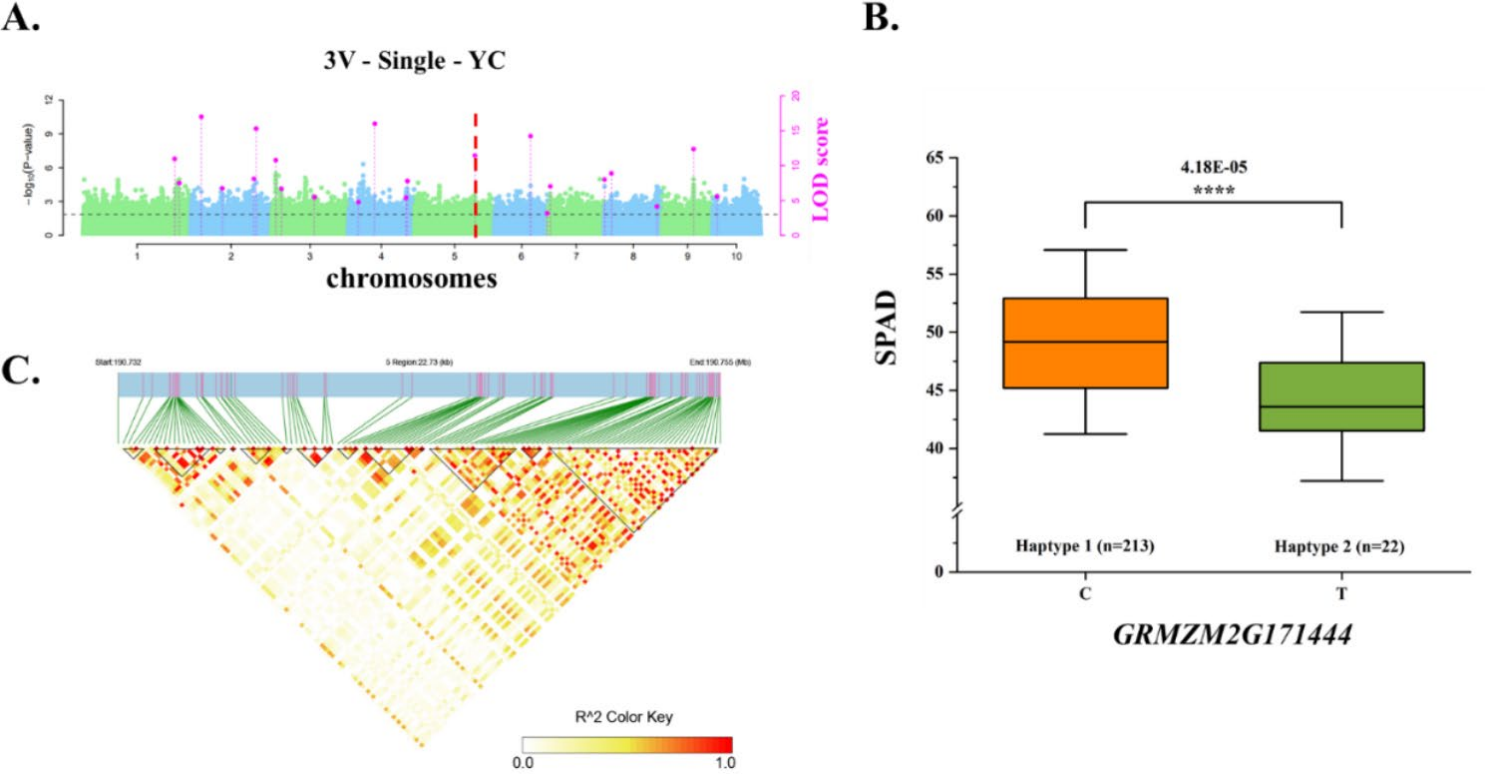
*GRMZM2G171444* affects CC-related traits. **(A)** Manhattan plot of the SPAD values for YC. The line represents the threshold LOD score ≥ 3.0. **(B)** Differences in the CC-related traits of haplotypes 1 and 2. **(C)** R^2^ values for all SNPs in the QTL of the significant QTN

**Fig. 9:**
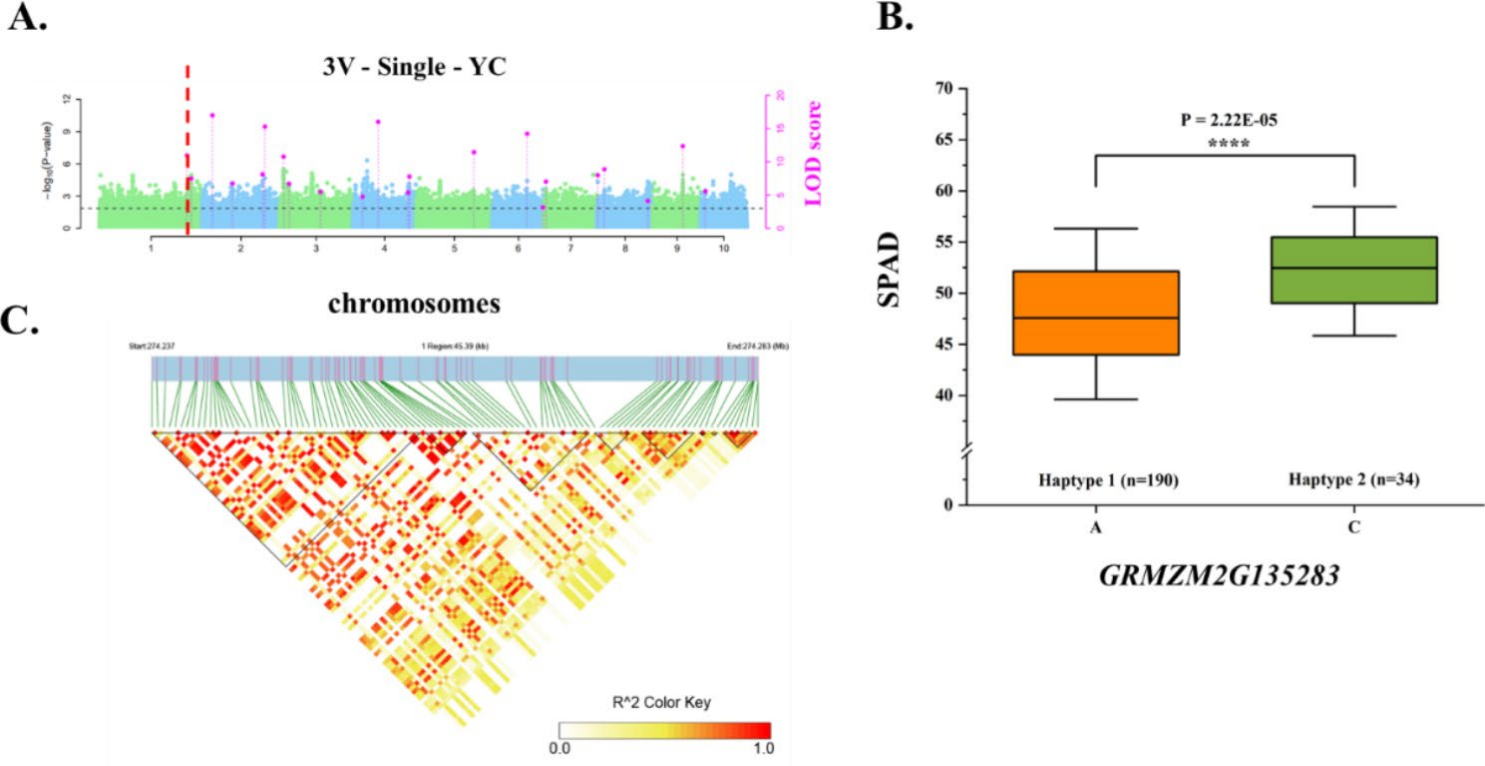
*GRMZM2G135283* affects CC-related traits. **(A)** Manhattan plot of the SPAD values for YC. The line represents the threshold LOD score ≥ 3.0. **(B)** Differences in the CC-related traits of haplotypes 1 and 2. **(C)** R^2^ values for all SNPs in the QTL of the significant QTN

We performed a haplotype analysis of the key candidate genes (Table S6). The *p*-values for the phenotypic differences associated with QTNs were less than 0.01 for chr3.S_17284190 (LOD = 6.7012, *p*-value = 2.77 × 10^− 8^) and chr3.S_168368384 (LOD = 15.2228, *p*-value = 5.60 × 10^− 17^) within *GRMZM5G839422* and *GRMZM2G017077* respectively. Whereas they were less than 0.001 for chr5.S_215364939 (*p*-value = 6.40 × 10^− 7^) and less than 0.0001 for the remaining eight QTNs. Accordingly, the phenotypic differences related to the SNPs in the 11 key candidate genes were all extremely significant (Figure S3, Figs. 7B, 8B and 9B). Next, we extracted all of the polymorphic loci within the QTLs containing the significant QTNs of the key candidate genes for the LD analysis (Figure S4). There was a strong linkage relationship between the significant QTNs of several candidate genes and the polymorphic sites (Fig. 8 C, 9 C). The linkage relationship was especially strong for *GRMZM2G110408*, which was detected using SUPER (BLUP and HB) and FarmCPU (HB) (Fig. 7).

## Discussion

Chlorophyll is critical for photosynthesis. Changes in CC directly affect the leaf photosynthetic efficiency, which ultimately influences the crop yield [48–50]. Maize plants can efficiently use light energy and accumulate a large amount of dry matter through photosynthesis [51]. Hence, the maize yield is greatly affected by photosynthesis, but it is also considerably influenced by leaf senescence if chlorophyll is degraded [38]. A recent study demonstrated that adjusting the timing of maize leaf senescence can substantially modify the maize yield [52]. There has been extensive research on chlorophyll-related genes, especially those associated with leaf senescence [53–55]. However, compared with other plants, there have been relatively few related studies on maize. Because of the considerable interest in chlorophyll synthesis, the underlying mechanism is continually being clarified [56, 57], but the molecular mechanism regulating CC will need to be further analyzed. In addition, there is growing interest in stay-green traits among crop breeders [58, 59]. Therefore, studying the genetic basis of maize CC, identifying important genetic variants, and mining-related candidate genes are crucial for the genetic improvement of maize via breeding.

Genome-wide association studies have been conducted to elucidate the genetic basis of complex quantitative traits and to screen for genes related to agronomic traits [60, 61]. In the current study,

the normal distribution of CC indicate that it is jointly determined by multiple genetic variants (Figure S1), and its heritability is calculated to be 0.86 (Table S4). Next, MLM [25], MLMM [30], SUPER [31], FarmCPU [32], BLINK [33], and 3VmrMLM [34] were used to analyze the ear leaf CC of 290 maize inbred lines at 10 days after pollination. More specifically, a GWAS was completed using 1.25 million high-density markers and the CC (SPAD values) (Figure S5). Finally, 140 significant QTNs and 481 genes were identified (Table S1). There were 19 significant co-located QTNs, of which 10 were detected by at least two different models or methods, three were detected in at least two different environments, and six were identified by different models or methods in different environments (Table 2). The statistical analysis of the number of candidate genes detected by each model indicated 3VmrMLM and SUPER detected the most candidate genes. Moreover, co-located candidate genes were detected by all of the models, except for MLM, with 45 co-located genes revealed by the two 3VmrMLM methods (Figs. 3 and 4; Table 2). These results may be relevant for identifying genes significantly associated with CC. Relevant published reports and the functionally annotated *A. thaliana* and rice homologs were used to clarify the functions of the co-located genes (Table 2). The enriched GO terms and KEGG pathways were considered and 11 candidate genes were selected for the subsequent experiments (i.e., expression analysis, haplotype analysis, and LD analysis of candidate genes). On the basis of the results of these experiments, we identified *GRMZM2G110408*, *GRMZM2G171444*, and *GRMZM2G135283* as candidate genes related to the maize ear leaf CC. Of these genes, we suggest *ZmCCS3* may be the most worthwhile candidate gene for future investigations. Among the 11 key candidate genes, the GO analysis indicated *GRMZM5G839422* likely encodes a protein localized in chloroplasts (Table S3). A mutation to the *A. thaliana* homolog (*ALB3*) of *GRMZM5G839422* reportedly leads to a decrease in CC [42]. In the present study, *GRMZM5G839422* was most highly expressed in the mature leaf stage (Fig. 6, Table S5). The GO terms assigned to *GRMZM2G005848* suggested the encoded protein is also associated with chloroplasts. Consistent with this observation, the *A. thaliana* homolog of this gene encodes a chloroplast protein [43]. The *GRMZM2G017077* expression level was relatively high (Fig. 6, Table S5). Additionally, its *A. thaliana* homolog *VIPP1* encodes a multifunctional chloroplast protein that may affect chloroplast functions and CC [45]. The GO analysis of *GRMZM2G376595* and *GRMZM2G098420* indicated that these two genes contribute to leaf senescence (biological process) (Fig. 5A). Moreover, *GRMZM2G098420* encodes autophagy protein 5. The overexpression of *KHZ1*, which is the *A. thaliana* homolog of *GRMZM2G056920*, can significantly promote leaf senescence [39]. We propose that these three genes may be involved in chlorophyll degradation. Both *GRMZM2G045314* and *GRMZM2G093347* were among the co-located genes. The rice homolog of *GRMZM2G045314* is related to grain development (e.g., grain filling stage) [40]. Earlier research showed that a mutation to *APT1*, which is a homolog of *GRMZM2G093347*, enhances the accumulation of chlorophyll in leaves [41]. These functions are closely related to CC, implying these genes should be more precisely characterized in future studies.

In this study, *GRMZM2G171444* was more highly expressed than the other candidate genes in the mature leaf stage (Fig. 6, Table S5). A significant QTN (chr5.S_190752068) in this gene was detected by 3VmrMLM in YC, with a LOD score of 11.45, suggestive of its importance (Table S2, Fig. 8A). This significant QTN was used for a haplotype analysis [62], which revealed that the phenotypic difference between the two haplotypes of this gene was significant (4.18 × 10^− 5^) (Fig. 8B). The *A. thaliana* and rice homologs of this gene encoding a ribosome protein have not been identified, but the *A. thaliana* and rice databases suggest the homologs may be involved in the synthesis of chloroplast precursors. This possibility will need to be experimentally verified. The *GRMZM2G135283* candidate gene detected by 3VmrMLM in YC contained a significant QTN (chr1.S_274280041) with a LOD score of 10.9716 (Table S2, Fig. 9A). The phenotypes associated with the two haplotypes of this QTN differed significantly (2.22 × 10^− 5^) (Fig. 9C). Because the LD analysis detected a strong linkage relationship, we speculate that the candidate gene is highly correlated with chlorophyll traits (Fig. 9B). We also determined that *GRMZM2G135283* encodes a serine hydroxymethyltransferase. A mutation to the rice homolog (*OsSHM1)* of this gene does not affect seed germination, but the mutant leaves are less green and have a lower CC than the wild-type leaves. Additionally, this rice homolog is expressed in all examined tissues (i.e., root, stem, leaf, and young ear), but especially in the leaves [47]. In the current study, 3VmrMLM detected more noteworthy candidate genes than the other models. Finally, and most importantly, our findings imply that *ZmCCS3* should be examined more comprehensively in future investigations. In particular, this gene was identified on the basis of two co-located QTNs (chr9.S_110657959 and chr9.S_110659989). The *p*-values for chr9.S_110657959 in the BLUP and HB environments of SUPER were respectively 6.9 × 10^− 7^ and 2.5 × 10^− 7^, which differed from the corresponding *p*-value in the HB environment of FarmCPU (1.3E-06). The *p*-values for chr9.S_110659989 in the BLUP and HB environments of SUPER were respectively 1.7 × 10^− 6^ and 9.3 × 10^− 7^ (Fig. 7A, Table S2). Furthermore, chr9.S_110657959, which was detected by the two models (SUPER and FarmCPU), was selected as a significant QTN for the haplotype analysis. The significance of the phenotypic difference between the two haplotypes was the highest among the 11 candidate genes (2.92 × 10^− 10^) (Fig. 7B), with haplotype 1 detected as the favorable haplotype. Of the 290 maize materials included in this study, 210 were temperate lines and 80 were tropical/subtropical lines, with the latter accounting for 27.59% of the examined materials. 214 materials contained the haplotype, with tropical/subtropical materials accounting for 25.23% (54/214) of the total. The findings of this study suggest that CC might be related to the germplasm type. Specifically, germplasm from temperate regions may have a higher CC than germplasm from other regions. Moreover, the LD analysis indicated that chr9.S_110657959 in *ZmCCS3* had a strong linkage relationship with polymorphic sites (Fig. 7C), implying this gene might be highly correlated with CC. The *A. thaliana* homolog of this gene encodes a metalloproteinase localized in the thylakoid membrane, wherein it repairs PSII adversely affected by photoinhibition [37]. The functional annotation showed that the nucleoside triphosphatase encoded by *ZmCCS3* is involved in chlorophyll biosynthesis (Table S2). Overall, we identified 11 candidate genes encoding proteins with regulatory effects on CC. Furthermore, we propose that *ZmCCS3* is critical for the regulation of CC. The data generated in this study may provide the basis of future research conducted to improve high photosynthetic efficiency of maize and breed ideotype-based maize varieties suitable for commercial cultivation.

## Conclusions

Our study compared the results of six GWAS models (a single-locus model and five multi-locus models), screen candidate genes within the range of co-located QTNs, combine functional annotation, GO and KEGG analysis, mine 11 CC-related key candidate genes. Based on the haplotype and LD analysis results of these key genes, *GRMZM2G110408* (*ZmCCS3*) is considered worthy of further study. This finding broadens the understanding of the genetic basis of CC and may be relevant for the ideotype-based breeding of new maize varieties with high photosynthetic efficiency.

## Materials and methods

### Experimental materials and field cultivation

The association mapping panel used in this study, which consisted of 290 maize inbred lines (210 temperate lines and 80 tropical/subtropical lines), was derived from 540 inbred lines [62, 63] and was provided by Professor Yan Jianbing of Huazhong Agricultural University. All 290 maize inbred lines were grown at the Yuanyang Modern Agricultural Science and Technology Park of Henan Agricultural University (Yuanyang; N35°, E113°; i.e., YY), the XunXian Experimental Station of the Hebi Academy of Agricultural Sciences in Henan province (Hebi; N35°, E114°; i.e., HB), and the Cotton Seed Farm in Yongcheng, Henan (Yongcheng; N33°, E116°; i.e., YC) in the summer of 2019. Two replicates of a complete randomized block design were used. Specifically, each line was grown in two (4 m long) rows, with 67 cm between rows and 10 plants per row. Routine field management practices were applied during the cultivation of open-pollinated plants.

### Determination of CC

For 10 days after pollination, five plants per row were randomly selected to determine the daily SPAD value (i.e., CC) for the ear leaf at 9:00–11:30. Briefly, a hand-held SPAD instrument: SPAD-502Plus (i.e., Minolta corporation, Ltd., Osaka, Japan) was used to measure the CC at three points of the ear leaf. Each plant was analyzed three times (error was less than 5%), after which the average value was recorded as the leaf CC. Finally, the average CC of five plants was used as the ear leaf CC of the inbred lines for the general statistical analysis of the phenotype and the GWAS. The phenotyping raw data of the CC for the 290 maize inbred lines across different environments and the best linear unbiased predictor (BLUP) values was provided in Table S7.

### Data processing and analysis

Microsoft Excel 2021 was used for the general statistical analysis of the variance in the SPAD values among the ear leaves in different environments. The data were visualized using RStudio and Origin 2021 (https://www.originlab.com/2021). The corr function of R (version 4.2.2) was used to analyze the correlation between the SPAD values for different environments. The BLUP values for all materials in three environments were calculated using the MLM of lme4 in the R package [64, 65]. The BLUP values were also used for the general statistical analysis and the subsequent GWAS, which can reduce the prediction bias caused by the unbalanced data [65]. The broad-sense heritability of the SPAD value was determined using R and the following formula:

$${\varvec{H}}^{2}={\varvec{\delta }}_{\varvec{G}}^{2}/\left[{\varvec{\delta }}_{\varvec{G}}^{2}+({\varvec{\delta }}_{\varvec{G}\varvec{E}}^{2}/\varvec{n}\right)+{\varvec{\delta }}_{\varvec{e}}^{2}/\left(\varvec{n}\varvec{r}\right)]$$

where ${\delta }_{G}^{2}$ is the genotypic variance, ${\delta }_{GE}^{2}$ is the variance in the genotype-by-environment interaction, ${\delta }_{e}^{2}$ is the error variance, $r$ is the number of replicates in an environment, and $n$ is the number of environments.

### Genome-wide association study

The genotype data obtained from the Maizego database (http://www.maizego.org/Resources.html) consisted of 1.25 million SNP (B73_RefGen_v2) that covered the whole maize genome, with a minimum allele frequency ≥ 0.05 [63]. Here, six models were implemented for GWAS, which included a single-locus model: Mixed Linear Model (MLM) and five multi-locus models, namely, Bayesian-information and Linkage-disequilibrium Iteratively Nested Keyway (BLINK), Multiple Loci Mixed Linear Model (MLMM), Fixed and random model Circulating Probability Unification (FarmCPU), Settlement of MLM Under Progressively Exclusive Relationship (SUPER) and 3 Variance-component multi-locus random-SNP-effect Mixed Linear Model (3VmrMLM). Specifically, MLM was implemented using TASSEL 5.0, whereas BLINK, MLMM, SUPER, and FarmCPU were implemented using “GAPIT” in the R package. The detected SNPs were referred to as lead SNPs. To determine whether there were false positives or negatives, Quantile–Quantile (QQ) plots for the five models (MLM, BLINK, MLMM, FarmCPU, and SUPER) were compared [66]. The LD among SNP markers was considered and the commonly used genome-wide threshold for detecting significant SNP–trait associations The suggested *p*-value (1.79 × 10^− 6^; 1/En) and − log_10_(*p*-value) ≥ 5.75 were calculated using a reported En (557,894) [67] after the quality control step.

The recently published 3VmrMLM method was implemented using the IIIVmrMLM software [34] from the GitHub website (https://github.com/YuanmingZhang65/IIIVmrMLM). The main-effect QTNs and QEIs in this model were detected using “Single_env” and “Multi_env”, with the following parameters: SearchRadius = 50; svpal = 0.01; and LOD score ≥ 3. Moreover, Manhattan and QQ plots were generated using the default parameters of “CMplot” in the R package (https://github.com/YinLiLin/R-CMplot) and 3VmrMLM.

### Analyses of candidate genes

For each QTN, a 100 kb interval (50 kb upstream and downstream ot the significant SNP) was defined as a QTL, where the LD decay distance was approximately 50 kb in the association mapping panel, and the candidate genes within all QTLs were searched. We sorted and summarized the candidate genes in the corresponding QTL among the models, methods, and environments and then analyzed the homologs of these candidate genes in *A. thaliana* and rice (*Oryza sativa*) using MaizeGDB (http://www.maizegdb.org), NCBI (www.ncbi.nlm.nih.gov), RiceData (https://ricedata.cn), and Phytozome v13 (https://phytozome-next.jgi.doe.gov). The candidate genes related to CC were then functionally annotated.

The Gene Ontology (GO) analysis and the Kyoto Encyclopedia of Genes and Genomes (KEGG) pathway analysis of the candidate genes were performed using OmicShare tools (https://www.omicshare.com/tools) [68]. The principle is as follows: first, genes obtained from the GWAS results, which had unknown functions, were excluded from further analysis. The remaining genes were mapped to corresponding GO database terms (http://www.geneontology.org/). enabling us to assign specific GO terms to each gene. Subsequently, the number of genes associated with each GO term was computed, which facilitated the creation of a gene list and allowed us to determine the frequency of genes linked to each GO function.

To identify significantly enriched GO entries among differentially expressed genes compared to the entire genome background, we employed a hypergeometric test. This statistical test served to evaluate whether the observed frequency of genes associated with a particular GO term was significantly higher than what would be expected by chance alone. Through this analysis, we were able to pinpoint GO terms that exhibited noteworthy enrichment among the differentially expressed genes. The formula utilized for the hypergeometric test is as follows [69]:

$$P = 1 - \sum\limits_{i = 0}^{m - 1} {\left(_i^M \right)\frac{{\left(_{n - i}^{N - M}\right )}}{{\left(_n^N \right)}}}$$

where, P is p-value, represents the probability of observing i or more genes associated with a specific GO term, N is the number of genes with GO annotation in all Unigene; n is the number of differentially expressed genes in N; M is the number of genes annotated for a specific GO term in all Unigene; m is the number of differentially expressed genes annotated as a specific GO term. By applying this statistical test, we were able to identify GO entries that were significantly enriched among the differentially expressed genes, providing valuable insights into the functional implications of the observed gene expression changes.

The p-value was set to ≤ 0.05. A Gene Ontology (GO) term that satisfied this condition was defined as a GO term with significant enrichment in differentially expressed genes. Regarding the KEGG analysis [112], the formula for determining significance is similar to that of GO. The formula is as follows: N represents the total number of genes (background genes). n represents the number of differential genes (target genes). M represents the number of occurrences of a specific pathway in all genes. If the p-value is ≤ 0.05, the pathway is considered significantly enriched in differentially expressed genes.

### Linkage disequilibrium analysis

The LD analysis was performed using all SNPs within the QTL containing significant QTNs. The heatmaps of the LD were constructed using LDBlockShow (Dong et al., 2021), which is available online (https://github.com/BGI-shenzhen/LDBlockShow).

### Haplotype analysis

The SNP haplotype analysis was performed for the candidate genes most likely related to CC after the comprehensive analysis. We utilize significant QTN for dividing into two haplotypes based on their genotype, which detected the candidate gene. The SPAD values for the environments in which the significant QTN of the candidate genes were detected were used as the phenotypic data. And t-test was performed on the phenotypic data of the two haplotypes to compare whether there were significant differences between the two haplotypes, which were plotted using Origin 2021(https://www.originlab.com/2021).

### Analysis of candidate gene expression patterns

To analyze candidate gene expression patterns, the expression data for the different samples in B73 available online (http://www.zeamap.com/) were compared. The heatmap of the FPKM-based expression levels for the key candidate genes was drawn using the Python package seaborn (https://seaborn.pydata.org/index.html).

## Electronic supplementary material

Below is the link to the electronic supplementary material.

**Additional File 1: Figure S1.** Normal distribution of CC (SPAD value) in different environments. **Figure S2.** QQ plots of the chlorophyll content (CC) for five models in different environments. **Figure S3.** Differences in the chlorophyll content (CC) associated with key candidate genes. **Figure S4.** R2 values for all SNPs in the QTL of the significant QTN. **Figure S5.** Manhattan plots of the CC for six models in different environments or involving different methods.

**Additional File 2: Table S1.** Variance in the chlorophyll content (SPAD value) in three environments (HB, YY, and YC). **Table S2.** Candidate genes detected by the six GWAS models. **Table S3.** Results of the GO and KEGG analyses of key candidate genes. **Table S4.** Summary of the variance in heritability. **Table S5.** Expression patterns of key candidate genes in different tissues. **Table S6.** Comparison of the haplotypes of key candidate genes in terms of the chlorophyll content (CC) of 290 inbred lines. **Table S7.** Phenotypic data of CC (SPAD values).

## Data Availability

The datasets included in this study are available in an online repository (https://www.ebi.ac.uk/eva/) under accession number PRJEB56161. Or can be downloaded in the Genotypic Data section of website (http://www.maizego.org/Resources.html) called [1.25 M with 540 sizes].

## Abbreviations

CC: Chlorophyll Content
SPAD: Soil-Plant Analysis Development
QTN: Quantitative trait nucleotides
QTL: Quantitative trait locus
MLM: Mixed Linear Model
BLINK: Bayesian-information and Linkage-disequilibrium Iteratively Nested Keyway
MLMM: Multiple Loci Mixed linear Model
FarmCPU: Fixed and random model Circulating Probability Unification
SUPER: Settlement of MLM Under Progressively Exclusive Relationship
3VmrMLM: 3 Variance-component multi-locus random-SNP-effect Mixed Linear Model
LD: Linkage Disequilibrium
GO: Gene Ontology
KEGG: Kyoto Encyclopedia of Genes and Genomes
