# Supplementary material for: Genetic dissection of maize (Zea mays L.) chlorophyll content using multi-locus genome-wide association studies
Source: BMC Genomics. 2023 Jul 10;24:384. doi: 10.1186/s12864-023-09504-0 (PMC10332058; doi:10.1186/s12864-023-09504-0)
Supplement: Supplementary file 1 — Additional File 1: Figure S1. Normal distribution of CC (SPAD value) in different environments. Figure S2. QQ plots of the chlorophyll content (CC) for five models in different environments. Figure S3. Differences in the chlorophyll content (CC) associated with key candidate genes. Figure S4. R2 values for all SNPs in the QTL of the significant QTN. Figure S5. Manhattan plots of the CC for six models in different environments or involving different methods. [file 12864_2023_9504_MOESM1_ESM.pdf]

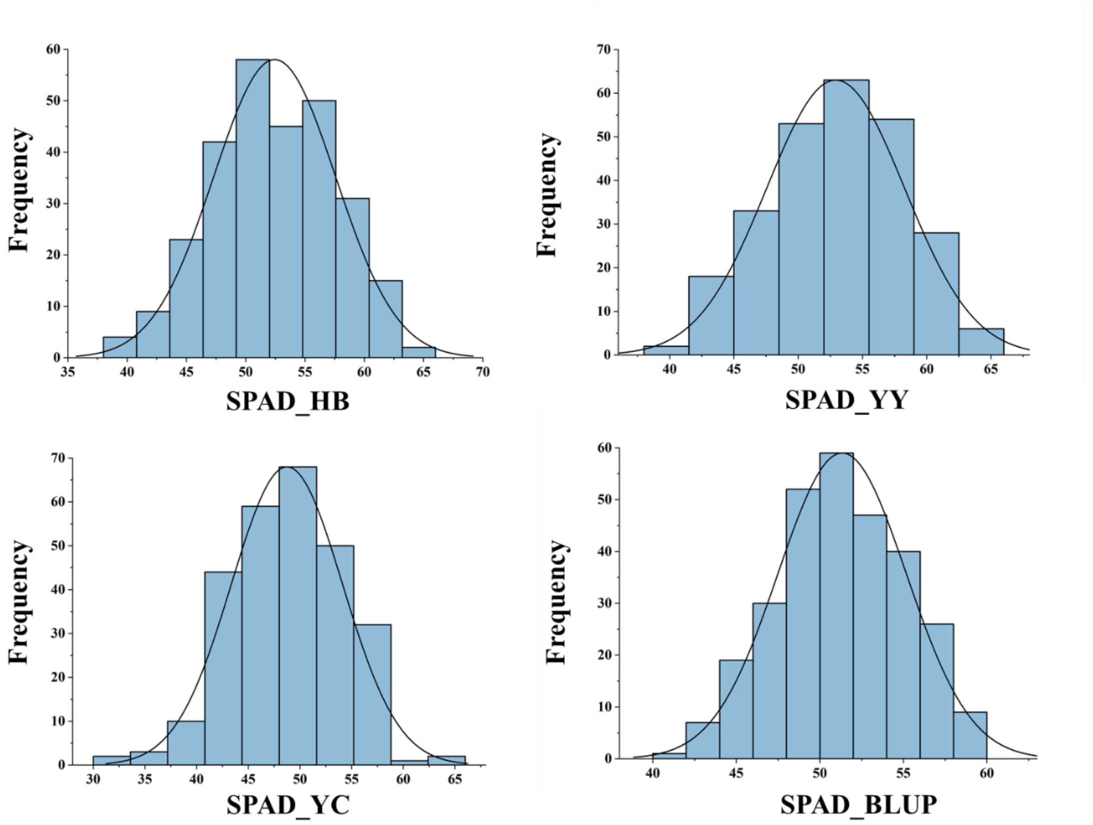

Figure S1. Normal distribution of CC (SPAD value) in different environments.

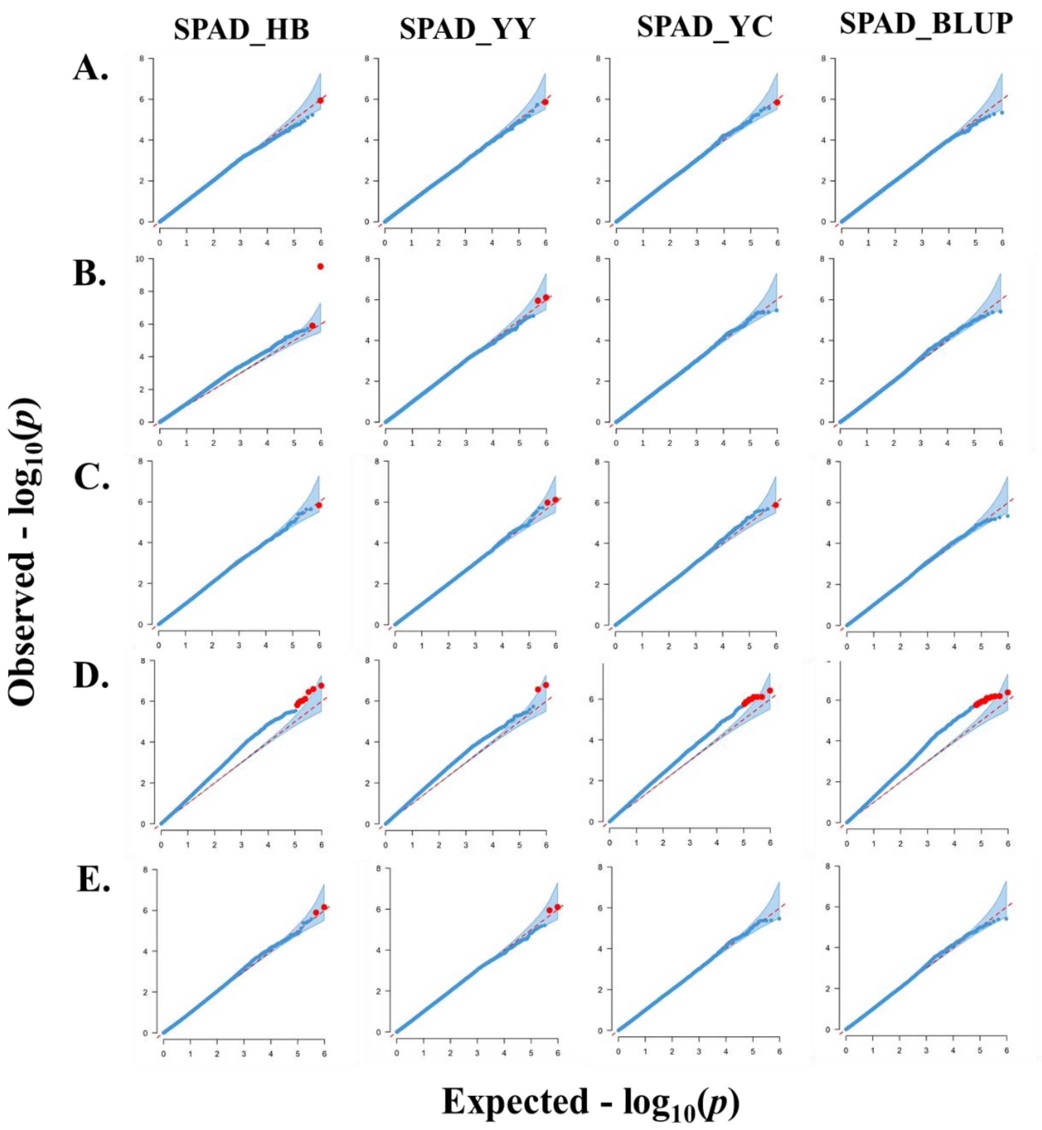

Figure S2. QQ plots of the chlorophyll content (CC) for five models in different environments. (A-E) represent the five models of MLM, BLINK, MLMM, SUPER, and FarmCPU, respectively.

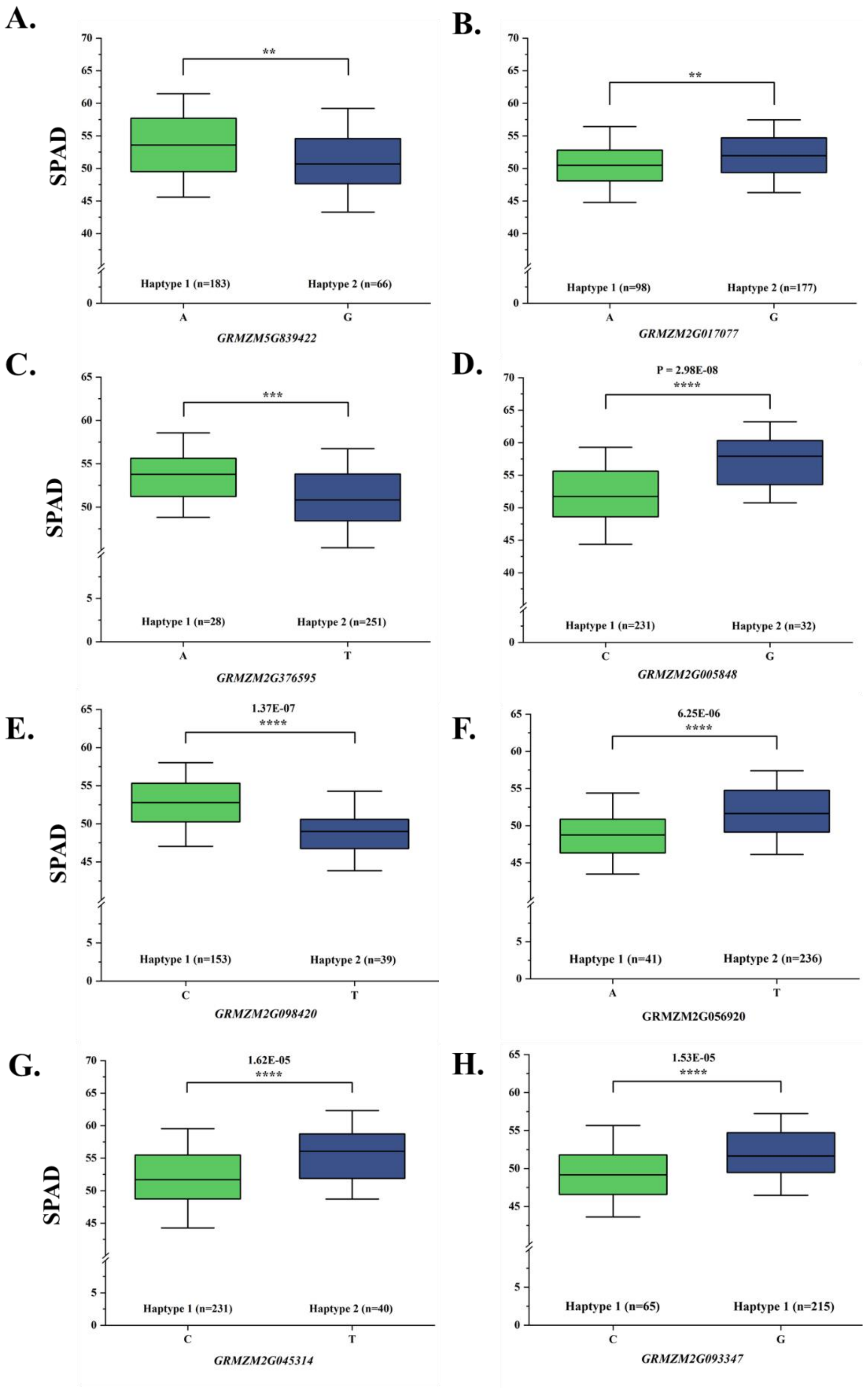

Figure S3. Differences in the chlorophyll content (CC) associated with key candidate genes. (A) *GRMZM5G839422*. (B) *GRMZM2G017077*. (C) *GRMZM2G376595*. (D) *GRMZM2G005848*. (E) *GRMZM2G098420*. (F) *GRMZM2G056920*. (G) *GRMZM2G045314*. (H) *GRMZM2G093347*. *: $p < 0.05$, **: $p < 0.01$, ***: $p < 0.001$, ****: $p < 0.0001$. Whisker range: standard deviation (SD), the coefficient is 1.5 to set the parameters of the box plot.

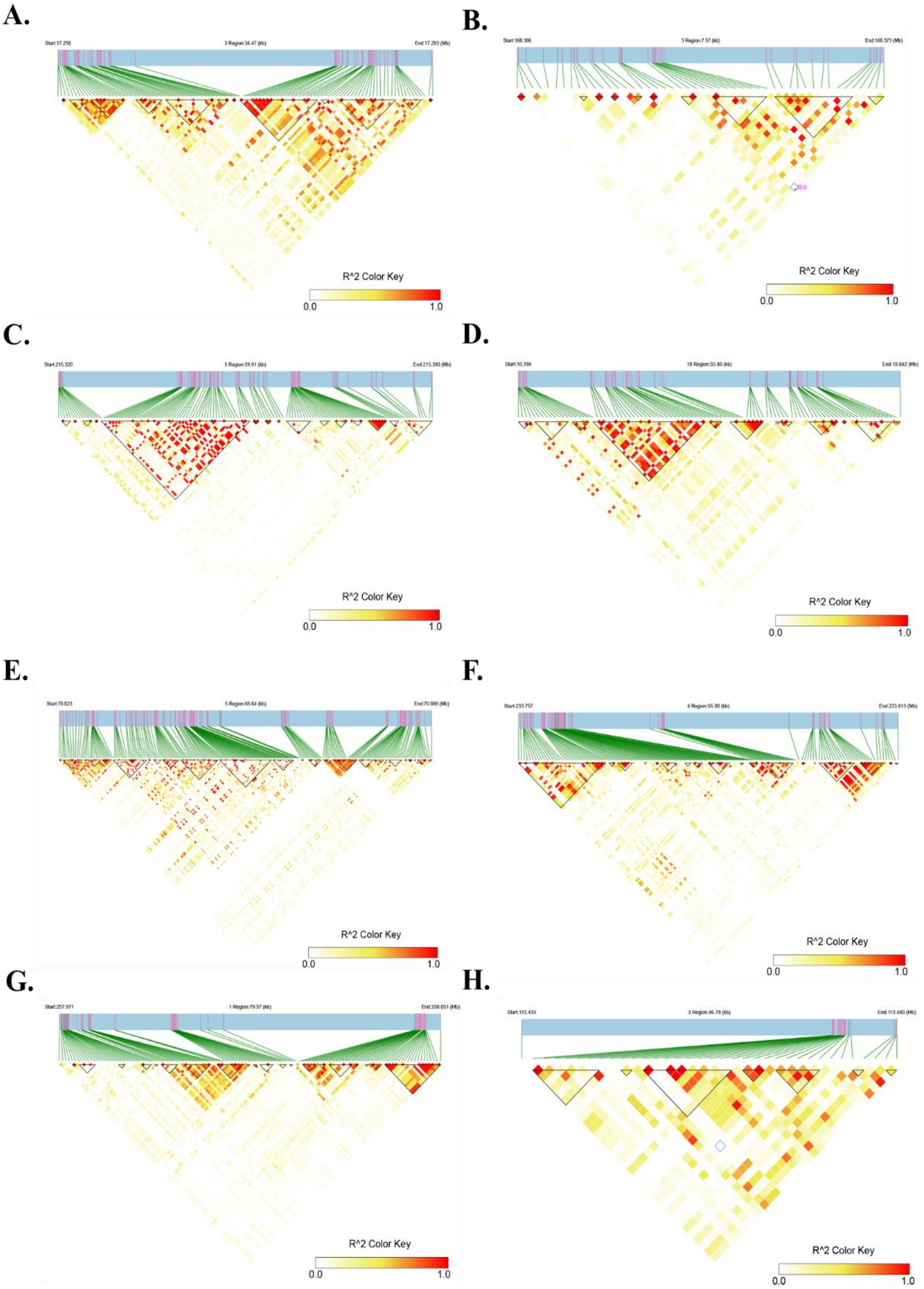

Figure S4. R^2 values for all SNPs in the QTL of the significant QTN. (A) *GRMZM5G839422*. (B) *GRMZM2G017077*. (C) *GRMZM2G376595*. (D) *GRMZM2G005848*. (E) *GRMZM2G098420*. (F) *GRMZM2G056920*. (G) *GRMZM2G045314*. (H) *GRMZM2G093347*.

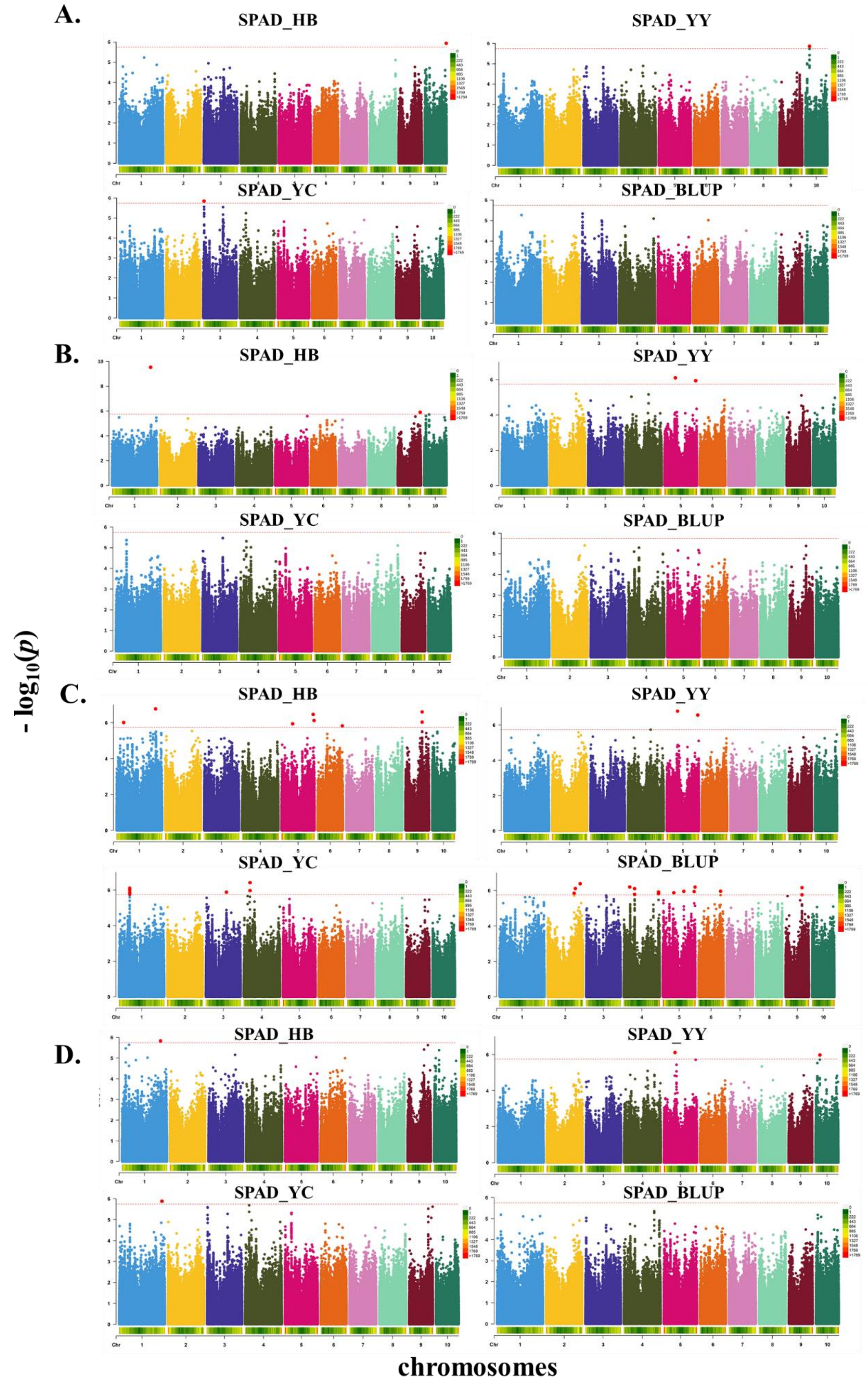
A.
SPAD_HB
SPAD_YY
SPAD_YC
SPAD_BLUP
B.
SPAD_HB
SPAD_YY
SPAD_YC
SPAD_BLUP
C.
SPAD_HB
SPAD_YY
SPAD_YC
SPAD_BLUP
D.
SPAD_HB
SPAD_YY
SPAD_YC
SPAD_BLUP
- log10(p)
chromosomes

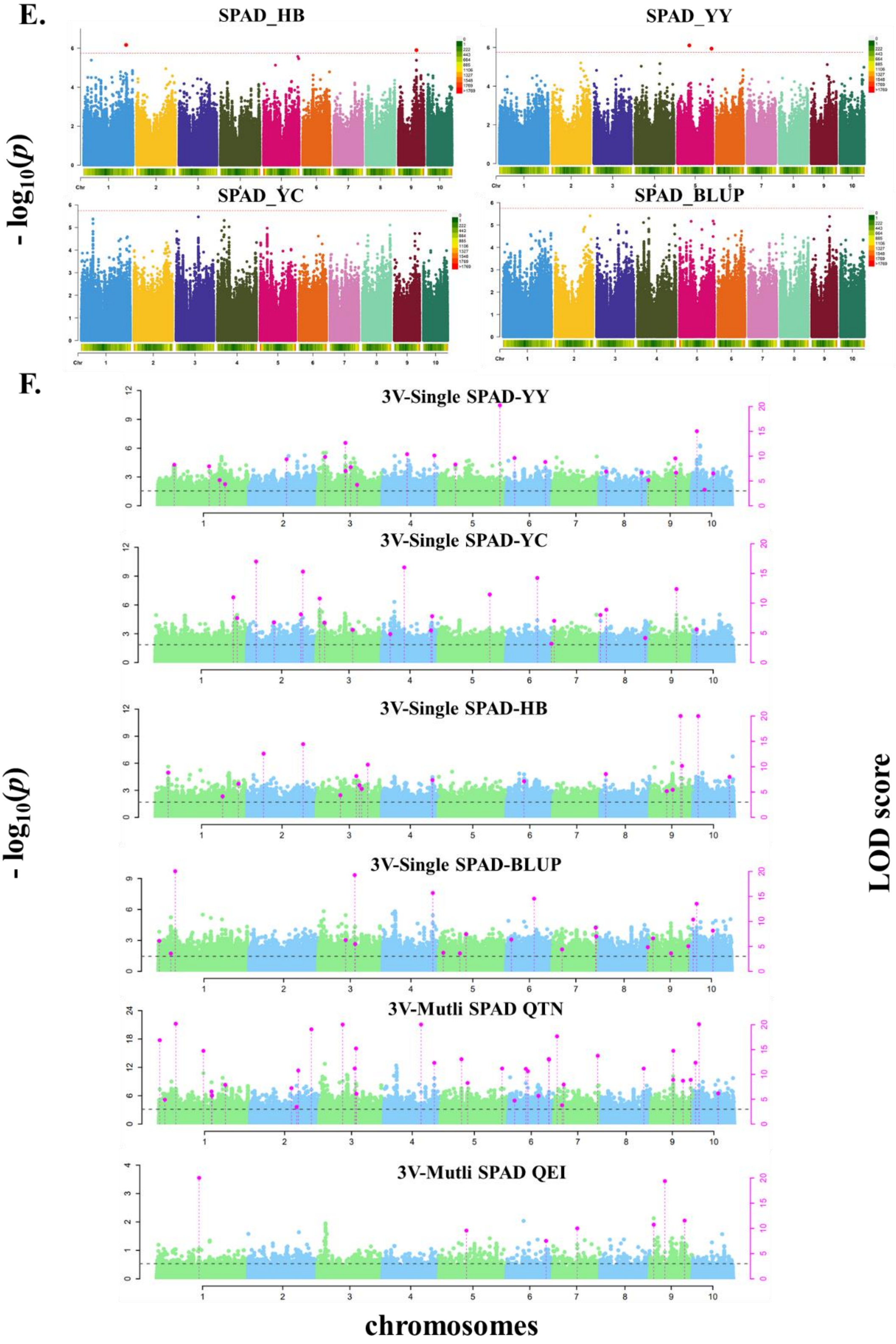

Figure S5. Manhattan plots of the CC for six models in different environments or involving different methods. (A-F) represent the six models of MLM, BLINK, SUPER, MLMM, FarmCPU and 3VmrMLM, respectively.
